# Impact of Bivalent BA.4/5 BNT162b2 COVID-19 Vaccine on Acute Symptoms, Quality of Life, Work Productivity and Activity Levels among Symptomatic US Adults Testing Positive for SARS-CoV-2 at a National Retail Pharmacy

**DOI:** 10.3390/vaccines11111669

**Published:** 2023-10-31

**Authors:** Manuela Di Fusco, Xiaowu Sun, Laura Anatale-Tardiff, Alon Yehoshua, Henriette Coetzer, Mary B. Alvarez, Kristen E. Allen, Thomas M. Porter, Laura Puzniak, Santiago M. C. Lopez, Joseph C. Cappelleri

**Affiliations:** 1 Pfizer, Inc., New York, NY 10001, USAjoseph.c.cappelleri@pfizer.com (J.C.C.); 2 CVS Health, Woonsocket, RI 02895, USAlaura.anatale-tardiff@bluehealthintelligence.com (L.A.-T.); henriette.coetzer@bluehealthintelligence.com (H.C.)

**Keywords:** SARS-CoV-2, BA.4/5 BNT162b2, bivalent, COVID-19, COVID-19 symptoms, HRQoL, humanistic, quality of life, WPAI, PROMIS Fatigue

## Abstract

Evidence on the impact of COVID-19 vaccination on symptoms, Health-Related Quality of Life (HRQoL) and Work Productivity and Activity Impairment (WPAI) is scarce. We analyzed associations between bivalent BA.4/5 BNT162b2 (BNT162b2) and these patient-reported outcomes (PROs). Symptomatic US adults testing positive for SARS-CoV-2 were recruited between 2 March and 18 May 2023 (CT.gov NCT05160636). PROs were assessed using four questionnaires measuring symptoms, HRQoL and WPAI (a CDC-based symptom survey, PROMIS Fatigue, EQ-5D-5L, WPAI-GH), from pre-COVID to Week 4 following infection. Multivariable analysis using mixed models for repeated measures was conducted, adjusting for several covariates. The study included 643 participants: 316 vaccinated with BNT162b2 and 327 unvaccinated/not up-to-date. Mean (SD) age was 46.5 years (15.9), 71.2% were female, 44.2% reported prior infection, 25.7% had ≥1 comorbidity. The BNT162b2 cohort reported fewer acute symptoms through Week 4, especially systemic and respiratory symptoms. All PROs were adversely affected, especially at Week 1; however, at that time point, the BNT162b2 cohort reported better work performance, driven by less absenteeism, and fewer work hours lost. No significant differences were observed for HRQoL COVID-19 negatively impacted patient outcomes. Compared with unvaccinated/not up-to-date participants, those vaccinated with bivalent BA.4/5 BNT162b2 reported fewer and less persistent symptoms and improved work performance.

## 1. Background

A growing body of evidence indicates that COVID-19 has profound implications on patients’ wellbeing and social function [1,2]. The multiorgan symptoms of SARS-CoV-2 infection have been associated with a decline in health-related quality of life (HRQoL), impairments in daily activities and the ability to work [3,4,5].

From the original monovalent vaccines to the bivalent vaccines, COVID-19 vaccination significantly impacted the global COVID-19 response. Evidence on the efficacy, safety and effectiveness of mRNA COVID-19 vaccination is extensive for the original monovalent formulation and is rapidly growing for the bivalent formulation as well [6,7,8]. The mRNA bivalent vaccines targeted both the original strain and BA.4/BA.5 Omicron lineages and were authorized for use as a single booster on 31 August 2022 to provide further protection against Omicron, replacing the original monovalent formulations [9].

The BNT162b2 COVID-19 vaccine has shown additive benefits beyond traditional endpoints [3,4,5]. In a previous nationwide study of symptomatic US outpatients, being boosted with the original monovalent BNT162b2 was associated with a significant reduction in the prevalence and persistence of both acute and long-term symptoms, and improved health-related quality of life, activity levels and work performance [4,5]. There is a dearth of such data for the bivalent formulation. This study sought to address these gaps and assess the burden of COVID-19 infection and the impact of the Pfizer–BioNTech COVID-19 vaccine (original and BA.4/5 bivalent) on symptoms, HRQoL, work productivity and activity impairment prior to through one month following SARS-CoV-2 infection.

## 2. Methods 

### 2.1. Study Design and Participants

This was a nationwide prospective patient-reported outcomes (PRO) survey-based study that leveraged a previously described design (clinicaltrials.gov NCT05160636) [4,5]. The source population consisted of individuals testing for SARS-CoV-2 at one of approximately 5000 CVS Health test sites across the United States (US). As part of the registration process for scheduling a SARS-CoV-2 test at a CVS Health site, individuals are required to complete a screening questionnaire including demographics, symptoms, comorbidities and COVID-19 vaccination status. The screening variables, reverse transcription–polymerase chain reaction (RT-PCR) test and rapid antigen test results are loaded in an analytic dataset, where ~80–90% of test results are reported within 2–3 days from the date of the test appointment. Leveraging this analytic platform, this study was designed as a prospective survey-based patient-reported outcomes study targeting adults 18 years of age and older with a positive RT-PCR or rapid antigen test result and self-reporting at least one acute COVID-19 symptom. The study excluded asymptomatic patients. The individuals meeting the inclusion and exclusion criteria were emailed an invitation as soon as their test results became available, no later than 4 days from testing and were recruited between 2 March 2023 and 18 May 2023 during the predominance of XBB Omicron sub-lineage circulation (Supplemental Figure S1). The email invitation directed the potential participants to a dedicated e-consent website to learn about the study, the survey schedule and to sign an informed consent. To encourage participation, email reminders were sent throughout the study follow-up period, and the survey was voluntary and anonymous. To minimize data missingness, respondents could not skip any surveys. Participants could discontinue participation from the research study at any time.

### 2.2. Study Cohorts

**Figure 1:**
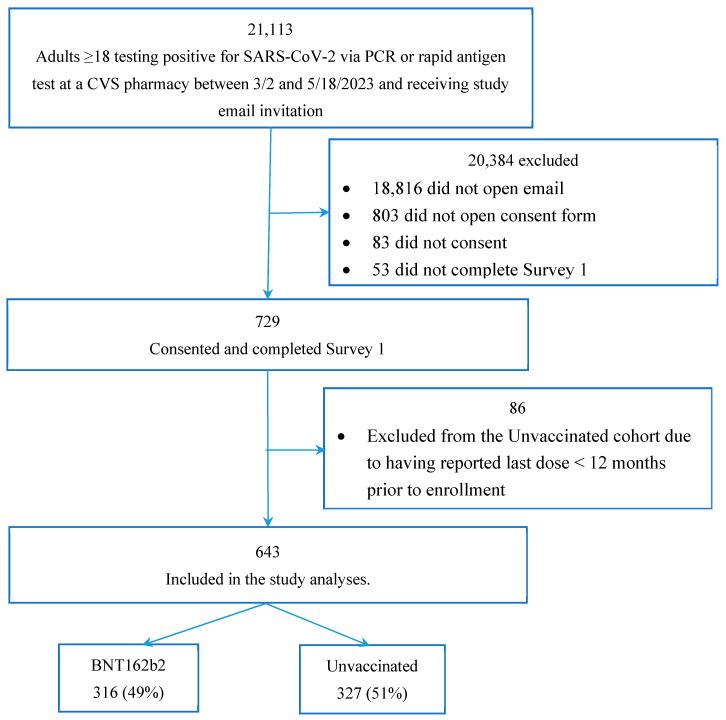
Study participant flowchart.

At enrollment, we categorized the study participants based on their pre-infection COVID-19 vaccination history. The study population for these analyses included two mutually exclusive cohorts: a “Bivalent” and an “Unvaccinated” cohort (Figure 1). Participants were included in the bivalent cohort if in the pre-test screening questionnaire they reported a date of 1 September 2022 or later [9] for their most recent dose of the Pfizer–BioNTech COVID-19 vaccine (original and BA.4/5 bivalent). From this date, the bivalent was the only formulation available and authorized in the US [9]. Participants were considered unvaccinated if they did not report any COVID-19 vaccine prior to testing or if they reported being primed (fully vaccinated) with their last monovalent dose received over 12 months before enrollment, the time at which their vaccine-induced immunity was assumed to have waned completely [10]. As such, this cohort comprised both unvaccinated and not up-to-date participants. For simplicity, this cohort is defined as “Unvaccinated” and this term is used throughout this report. To confirm vaccination status, participants’ subsequent responses to vaccination date questions were compared with their index responses (at time of registering for testing); if responses did not match, the information was queried and adjudicated, and the latest information was used.

### 2.3. Baseline Characteristics and Patient-Reported Outcome Measures

The baseline characteristics of study participants were obtained via the CVS Health pre-test screening questionnaire, which comprised demographics, comorbidities, COVID-19 vaccination and COVID-19 infection history. The Social Vulnerability Index (SVI) was calculated based on zip codes, with a value of 0 representing the lowest level of vulnerability and a value of 1 representing highest vulnerability [11]. An additional questionnaire captured COVID-19 antiviral treatment use and changes in vaccination and infection status at each study time point. The study outcomes included symptoms, HRQoL, fatigue, work productivity and activity impairment. These outcomes were assessed via validated PRO measures (respectively, EQ-5D-5L, PROMIS Fatigue 8a, WPAI:GH) and ad hoc questionnaires, at different time points (Day 3, Week 1, Week 2, Week 4).

### 2.4. Acute Symptoms

Using the CDC list of acute symptoms [12] as reference, the study assessed the presence of 12 symptoms at time of testing, Week 1, 2 and 4, across 3 categories: (1) systemic (including fever, chills, muscle or body aches, headaches, fatigue); (2) respiratory (including shortness of breath or difficulty breathing, cough, sore throat, new/recent loss of taste or smell); (3) gastrointestinal (GI) (including nausea or vomiting, diarrhea). Symptom trajectories were reported as symptom point prevalence at each time point of the study. The pre-test screening questionnaire captured symptoms when participants scheduled their test, while an ad hoc questionnaire including the same symptoms was administered at Weeks 1 and 2. At Week 4, an ad hoc questionnaire was administered listing the 30 long-COVID symptoms including those from the CDC [13]. A total of 11 symptoms in this list matched the list of acute symptoms (all but “congestion or runny nose”) and were assessed and reported in these analyses.

### 2.5. Health-Related Quality of Life (HRQoL)

The study assessed HRQoL via the validated EQ-5D-5L questionnaire [14] that subjects were asked to complete at enrollment (Day 3), Week 2 and Week 4. On the day of enrollment, the consented participants completed the questionnaire twice, using two different versions: one was the standard version in present tense to assess current HRQoL, and the other was a modified version with questions in past tense to retrospectively assess pre-COVID-19 HRQoL. To minimize responder bias, the order of administration of the two versions was random. Five dimensions of EQ-5D-5L at each time point were converted into the Utility Index (UI) using the US-based weights established by Pickard et al. [15]. UI and visual analogue scale (VAS) scores were compared among cohorts and across assessment times. Lower scores for both EQ VAS and UI correspond to lower overall self-reported health-related quality of life. 

### 2.6. Work Productivity and Activity Impairment 

The Work Productivity and Activity Impairment General Health v2.0 (WPAI:GH) instrument was used to measure impairments in both paid and unpaid work [16,17]. Participants were asked to complete the survey at Week 1, Week 2 and Week 4. At Week 1, participants were asked to complete the questionnaire twice, with a first assessment referencing seven days prior to COVID-19 symptom onset, and a second assessment referencing the past seven days. Higher scores correspond to greater activity impairment and work productivity loss. Only participants who reported being employed were included for work productivity analyses. WPAI results were compared among cohorts and across assessment times. 

### 2.7. Fatigue

Fatigue was measured in the CDC-based symptoms questionnaire and also with the validated Patient-Reported Outcomes Measurement Information System (PROMIS) Fatigue 8a, a short-form fixed instrument comprising 8 of the 90-item PROMIS Fatigue item bank [18,19]. Participants were asked to complete the survey at Week 1, Week 2 and Week 4. At Week 1, participants were asked to complete the questionnaire twice, with a first assessment referencing seven days prior to COVID-19 symptom onset, and a second assessment referencing the past seven days. PROMIS uses T-scores, a type of standard score referenced to the US general population norms, which have a mean of 50 and standard deviation (SD) of 10. The raw summation score of 8 items was converted to standardized T-score based on the US population average [20]. Higher T-scores indicate worse fatigue [18].

## 3. Statistical Analysis

Means and standard deviations for continuous variables and frequency and percentages for categorical variables were used to summarize participant characteristics at baseline and outcomes at follow-up. T-tests and chi-square tests were used to test for continuous variables and categorical variables, respectively, to measure between-group differences. Fisher’s exact tests were used for 2-by-2 tables and Fisher–Freeman–Halton tests for r-by-c tables when an expected cell frequency was less than 5 [21,22]. All *p* values were two-sided. 

Mixed models for repeated measures (MMRM) were used to estimate the impact of vaccination on symptoms, HRQoL and WPAI over time [23]. The models included variables for time, vaccination status and interaction of time by vaccination status, as well as covariates of participant pre-COVID-19 symptom onset score, sociodemographic characteristics (age, sex, regions, social vulnerability, race/ethnicity), variable for at least 1 comorbidity, previously tested positive for COVID-19, severity of acute illness (number of symptoms reported on index date) and prescription of Paxlovid. Assessment time was fitted as a categorical covariate and a repeated effect (repeated by subject) with unstructured covariance matrix. Least-square mean (LS mean) and standard errors of PRO scores for each time point of assessment were calculated. Per guidelines, no adjustment was made for missing data when scoring the EQ-5D-5L Utility Index (UI) [15] and WPAI [17]. All available data were included in the analysis. 

We calculated Cohen’s d to assess the difference in pre-COVID scores among patients vaccinated and unvaccinated, the magnitude of score change from baseline to each time point within each cohort, as well as the differences between cohorts (bivalent vs. unvaccinated) [24,25]. Specifically, within-cohort effect size (ES) was calculated as mean change from baseline to follow-up, divided by the standard deviation of change scores from baseline to follow-up [4]. Between-cohort ES was calculated as the difference in mean score between cohorts, divided by the pooled standard deviation of scores or, alternatively, the difference in mean changes from baseline between cohorts, divided by the pooled standard deviation of change scores [4]. Values of 0.2, 0.5, and 0.8 standard deviation (SD) units represent “small”, “medium”, and “large” effect sizes, respectively [4,24]. 

On an exploratory basis, the two cohorts were matched on the clinical and demographic variables that differed, as an interim step between the analyses of the raw data and the MMRM. Matching results are presented in Supplementary Material.

All analyses were conducted with SAS Version 9.4 (SAS Institute, Cary, NC, USA). The study followed the Strengthening the Reporting of Observational Studies in Epidemiology (STROBE) reporting guideline [26].

## 4. Results

### 4.1. Baseline Characteristics

A total of 21,113 eligible candidates who tested positive at a CVS Health test site were outreached. Of these, 643 consented and met the inclusion and exclusion criteria for the analyses: 316 (49.1%) received bivalent BA.4/5 BNT162b2 and 327 (50.1%) were unvaccinated (Figure 1). Compared with individuals in the CVS Health analytic dataset who did not participate in our study, the study sample was slightly older, over-represented by women and Caucasians, with slightly more comorbidities and lower SVI. The study sample reported a similar number of acute symptoms during the infection, although a higher proportion of those in the study reported cough, new loss of taste/smell, nausea, or vomiting, and a lower proportion reported diarrhea and muscle or body aches (Supplemental Table S1). 

Baseline sociodemographic characteristics of the participants are shown in Table 1. Overall, the mean (SD) age was 46.5 (15.9), 70.3% were female, 58.2% Caucasian, and 40.4% were from the Southern US. The sample was characterized by moderate social vulnerability (mean SVI: 0.446). Almost half (44.2%) reported a previous COVID-19 infection, 25.7% reported at least 1 comorbidity, and 24.3% reported being prescribed a COVID-19 antiviral for the current infection. Compared with unvaccinated, bivalent BA.4/5 BNT162b2 participants were comparable with respect to sex. However, they tended to be older, Caucasian, reside in the Southern US, have lower SVI, have more comorbidities and utilize COVID-19 antivirals. In the vaccinated group, mean (SD) time between bivalent vaccination and infection was 165 (SD: 45) days. At Week 4, 72 (22.8%) and 100 (30.6%) participants did not respond to the assigned surveys, respectively, in the bivalent and unvaccinated cohorts. After matching for age, race/ethnicity, SVI category, region, ≥1 comorbidity, and antiviral use, the patient characteristics between the two groups were balanced (Supplemental Table S2).

### 4.2. Acute Symptoms

At the time of testing, study participants reported a mean of 5.3 symptoms, with the most frequent being respiratory and systemic symptoms (Table 2). BA.4/5 BNT162b2 participants reported fewer overall acute COVID-19 symptoms than unvaccinated: a mean of 5.0 vs. 5.7 (*p* < 0.001). The proportions of systemic symptoms were lower in the BA.4/5 BNT162b2 cohort, driven by lower frequency of fever, chills, muscle or body aches and headaches. All the other symptoms were directionally less frequent in the BA.4/5 BNT162b2 cohort (Table 2). These results were generally consistent based on the (repeated measures) model and after matching (Table 2, Supplemental Table S3). Supplemental Figure S2 shows the prevalence of individual symptoms by exposure. Supplemental Figure S3 shows the prevalence of acute COVID-19 symptoms over time by category.

At Week 1, the number of symptoms halved, dropping to a mean of 2.6 (Table 2). The BA.4/5 BNT162b2 cohort had a numerically lower, although not statistically significant, mean number of symptoms (2.5 vs. 2.7, *p* = 0.129) driven by fewer systemic symptoms (fever, muscle or body ache, headache). The other symptoms were directionally less frequent in the BA.4/5 BNT162b2 cohort, except for congestion, runny nose, and diarrhea. These results were generally consistent after matching (Supplemental Table S3); the model-based results showed that vaccinated participants had less fever and headache (Table 2).

At Week 2, the mean number of symptoms dropped to 1.9 (Table 2). Except for loss of taste or smell, congestion or runny nose, and nausea or vomiting, symptoms were directionally less frequent in the BA.4/5 BNT162b2 cohort. These results were consistent based on the model (Table 2) and matching (Supplemental Table S3). 

At Week 4, the mean number of acute symptoms dropped to 0.9 (Table 2). BA.4/5 BNT162b2 participants reported fewer symptoms than unvaccinated (mean: 0.7 vs. 1.1, *p* = 0.002), driven by less systemic symptoms and respiratory symptoms. Fatigue and sore throat were less prevalent in the BA.4/5 BNT162b2 cohort. After matching, the point prevalence of muscle or body aches was also lower in the bivalent cohort (Supplemental Table S3). All the other symptoms were numerically less frequent in the BA.4/5 BNT162b2 cohort (Table 2). 

**Figure 2:**
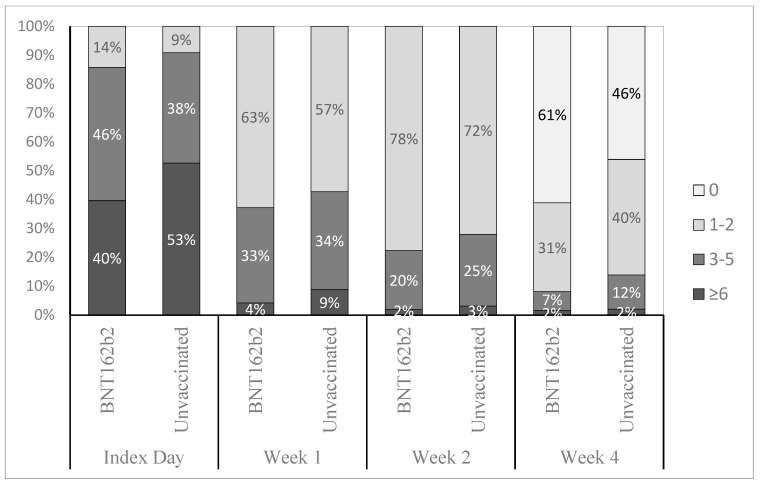
Distribution of study participants across ordinal categories of number of symptoms and vaccination status, across time points. Index day: time of testing.

When stratifying participants by ordinal categories of self-reported symptoms (0, 1–2, 3–5, 6+) and vaccination status, the BA.4/5 BNT162b2 cohort was characterized by lower proportions of participants with high symptom burden compared with unvaccinated across all time points and to a greater extent at the time of testing and Week 4 (Figure 2). 

### 4.3. Health-Related Quality of Life 

**Figure 3:**
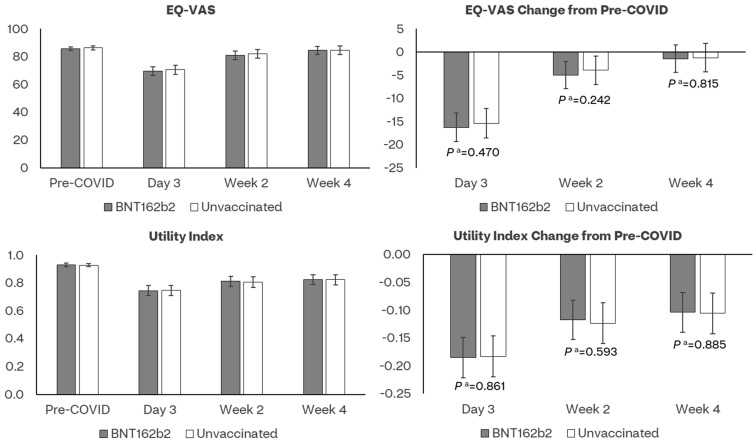
Least-square mean estimates of EQ−5D−5L and their changes from pre-COVID-19 baseline by assessment time and vaccination status. ^a^ *p* value of *t*-test comparing least-square mean score changes from baseline between BNT162b2 and Unvaccinated cohorts.

The mean pre-COVID-19 UIs did not differ between the bivalent BA.4/5 BNT162b2 and unvaccinated cohorts, respectively, 0.930 and 0.928 (*p* = 0.804) (Figure 3, Supplemental Table S4). COVID-19 had a detrimental effect on the HRQoL of participants, especially during Day 3. In both the BA.4/5 BNT162b2 and the unvaccinated cohorts, UIs were lower at Day 3, Week 2 and 4 relative to pre-COVID-19. While improvement was observed over time, neither the observed nor the model-based UI scores returned to pre-COVID levels at Week 4. The observed and model-based UIs were numerically higher in the BA.4/5 BNT162b2 cohort across all time points but were not significantly different from those in the unvaccinated cohort (Figure 3, Supplemental Tables S4 and S5). Mean pre-COVID-19 EQ-VAS scores were similar for the BA.4/5 BNT162b2 and unvaccinated cohorts, respectively, 85.8 and 86.3 (*p* = 0.581) (Figure 3, Supplemental Table S4). The observed and model-based UIs were numerically lower in the BA.4/5 BNT162b2 cohort across all time points and were not significantly different from those in the unvaccinated cohort (Figure 3). These results were generally similar post-matching (Supplemental Table S7).

### 4.4. PROMIS Fatigue

**Figure 4:**
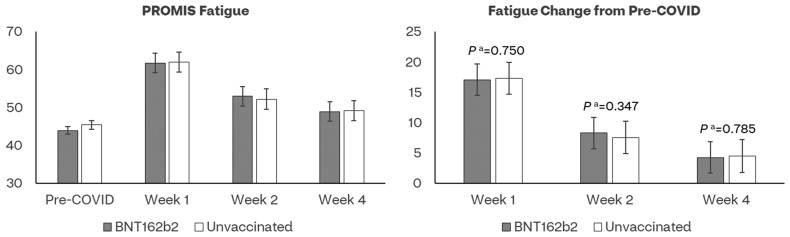
Least-square mean estimates of PROMIS Fatigue and their changes from pre-COVID-19 baseline by assessment time and vaccination status. ^a^ *p* value of *t*-test comparing least-square mean score changes from baseline between BNT162b2 and Unvaccinated cohorts.

The mean pre-COVID-19 baseline fatigue scores did not differ between the BA.4/5 BNT162b2 and unvaccinated cohorts, respectively, 44.0 and 45.4 (*p* = 0.065) (Figure 4, Supplemental Table S4). COVID-19 infection had a detrimental effect on fatigue of participants, especially at Week 1 (T-score = 60.2, indicating moderate fatigue). In both the BA.4/5 BNT162b2 and the unvaccinated cohorts, the observed and model-based scores were lower at all time points compared to pre-COVID-19. While improvement was observed over time, neither the observed nor the model-based scores returned to pre-COVID levels at Week 4 (Supplemental Tables S4 and S5). No significant differences were observed between the two cohorts. Results were generally similar post-matching (Supplemental Table S7).

### 4.5. Work Productivity and Activity Impairment

Approximately 74.1% of participants reported being employed at the time of testing (196 (62%) in the BA.4/5 BNT162b2 cohort and 224 (69%) unvaccinated), hence eligible to complete the absenteeism, presenteeism and work-productivity loss questions of WPAI:GH. 

**Figure 5:**
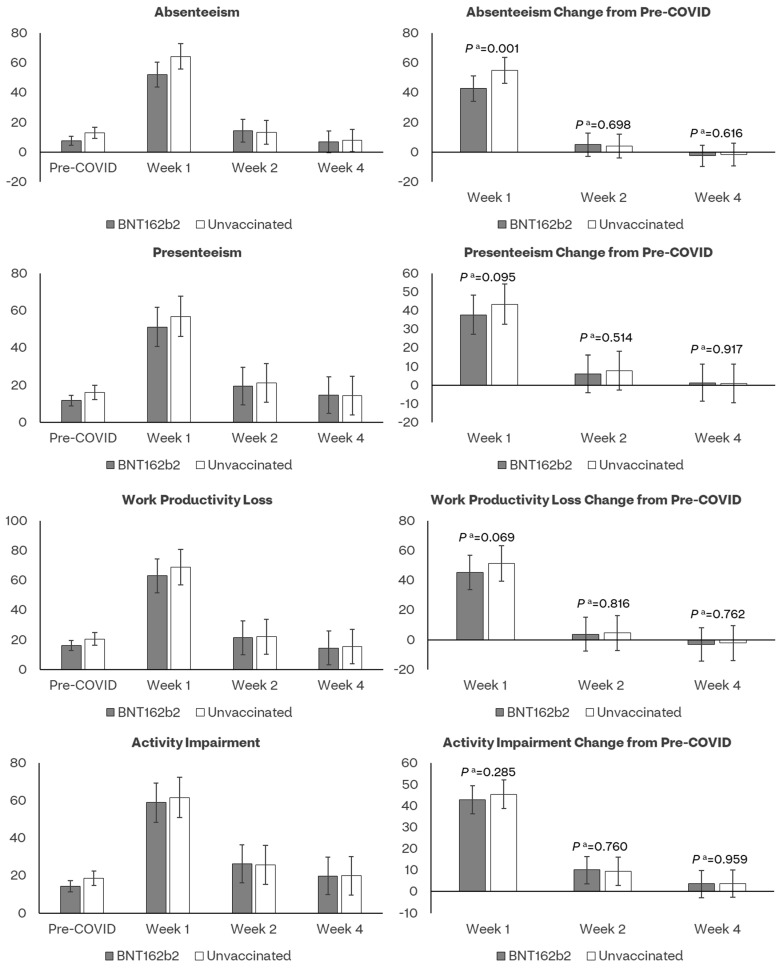
Least-square mean estimates of WPAI:GH scores and their changes from pre-COVID-19 baseline by assessment time and vaccination status. ^a^ *p* value of *t*-test comparing least-square mean score changes from baseline between BNT162b2 and Unvaccinated cohorts.

The mean pre-COVID-19 presenteeism and work productivity scores did not differ between the bivalent BA.4/5 BNT162b2 and unvaccinated cohorts, while absenteeism and activity impairment scores were slightly lower for the bivalent BA.4/5 BNT162b2 cohort (Supplemental Table S4). COVID-19 infection negatively affected all WPAI dimensions, especially during Week 1 (Figure 5, Supplemental Tables S4 and S5). 

The absenteeism score was lower among vaccinated than unvaccinated at Week 1 (52.0% versus 64.2%), with a moderate ES of 0.31. In both cohorts, absenteeism returned to levels comparable to pre-COVID at Week 2 (Figure 5, Supplemental Table S5). The presenteeism and work productivity scores returned to levels comparable to pre-COVID at Week 2 in both cohorts. The observed and model-based scores for these two WPAI domains were numerically lower among vaccinated, although not significantly different (Supplemental Tables S4 and S5). 

**Figure 6:**
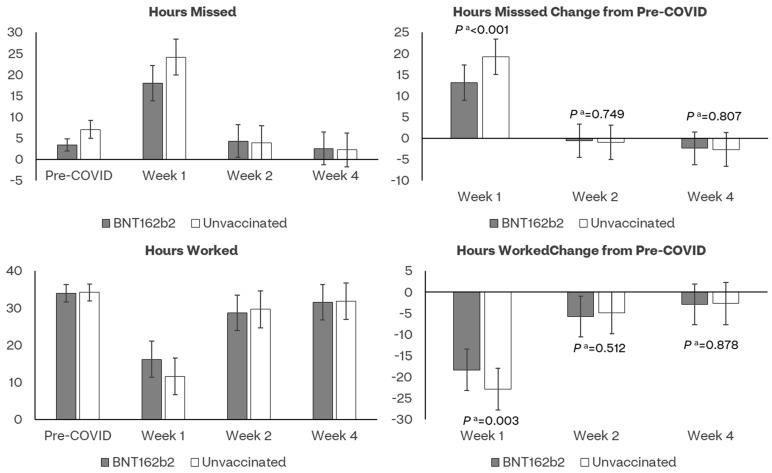
Least-square mean estimates of work hours lost and worked and their changes from pre-COVID-19 baseline by assessment time and vaccination status. ^a^ *p* value of *t*-test comparing least-square mean score changes from baseline between BNT162b2 and Unvaccinated cohorts.

The bivalent cohort reported significantly fewer work hours missed during Week 1 versus the unvaccinated cohort (LSE mean: 18.0 versus 24.2); consistently, they reported more work hours worked (LSE mean: 16.2 versus 11.7) (Figure 6, Supplemental Table S5). 

The nonwork-related activity impairment scores returned to levels comparable to pre-COVID at Week 4 in both cohorts; scores were numerically lower among vaccinated, although not significantly different (Figure 5). The WPAI results were generally similar after matching (Supplemental Table S7). The model parameter estimates are presented in Supplemental Table S6. 

## 5. Discussion

This nationwide study estimated the impact of the Pfizer–BioNTech BNT162b2 BA.4/5 bivalent vaccine on acute COVID-19 symptoms, Health-Related Quality of Life, Fatigue and Work Productivity and Activity Impairment among symptomatic adults testing positive for SARS-CoV-2 at a large national US pharmacy chain during circulation of the XBB 1.5 Omicron sub-lineage. Overall, the study found that COVID-19 infection had a detrimental effect on all patient outcomes, especially during Week 1, resulting in prolonged limitation of activities and of work productivity. However, the bivalent BA.4/5 BNT162b2 cohort was associated with significantly fewer and less persistent acute systemic and respiratory symptoms at the time of testing and Week 4. Across all time points, the BA.4/5 BNT162b2 cohort was characterized by lower proportions of participants with high symptom burden compared to unvaccinated/not up-to-date. These findings suggest that BA.4/5 BNT162b2 could alleviate the severity of infection, as measured by symptoms, and support patient recovery after SARS-CoV-2 infection. 

Our findings on acute symptoms are concordant with the results of our prior study [4] that employed a similar design to assess the impact of monovalent BNT162b2 on the same acute symptoms and PROs, recruiting subjects a year earlier, during the first quarter of 2022. The study populations shared key similarities in characteristics, frequency and prevalence of acute symptoms at time of testing, and pre-COVID EQ-5D-5L and WPAI scores. In both studies, the BNT162b2 COVID-19 vaccine was associated with fewer acute systemic symptoms at time of testing and fewer systemic and respiratory symptoms at Week 4, with some symptoms (fever, chills, loss of taste or smell, sore throat) nearing resolution among vaccinated. These findings indicate a consistent additive benefit for BNT162b2 beyond prevention of severe disease, even with a new formulation and with the emergence of new sub-lineages.

Differently from our prior study, in this study we used PROMIS Fatigue 8a and noted that the mean fatigue T-score at Week 1 (60.2) was comparable to that of rheumatoid arthritis (8a T-score: 58.6) [27]. At the Week 4 data cutoff, fatigue levels did not return to levels similar to pre-COVID US. These findings highlight the impact of COVID-19 on fatigue levels, which appear comparable to the effects of chronic debilitating diseases during Week 1 of infection. 

Additionally, during Week 1, the bivalent BA.4/5 BNT162b2 cohort reported less absenteeism and less impact on workplace performance: participants vaccinated reported 18.8 workhours missed versus 25.4 among unvaccinated. Although not statistically significant, activity impairment scores were numerically lower in the vaccinated cohort across all time points. 

Associations of bivalent BA.4/5 BNT162b2 with WPAI outcomes were more modest than those reported for the monovalent formulation, as they were observed during Week 1 only [4]. Also, we did not observe differences between the groups on HRQoL, differently from that observed for monovalent BNT162b2. Protection from COVID-19 bivalent vaccination has been observed to vary by Omicron sub-lineage, prior infection status, time since vaccination, time since prior infection, severity of infection and presence of risk factors [28,29]. Bivalent vaccination was targeted against BA.4/BA.5 Omicron sub-lineages and, while real-world evidence studies suggested cross-protection against successive sub-lineages, XBB was reported to be highly immune-evasive, triggering the need for a new, more closely matched, XBB vaccine formulation for 2023/2024 campaigns [30]. 

Considering that time since vaccination was relatively similar between the two studies (5–6 months), the more modest associations could be explained by the potential for reduced and less durable protection of the bivalent vaccine against XBB. In addition, although we controlled for prior infection, we could not measure time since prior infection. Accordingly, we could not sufficiently assess levels of immunity. Evidence has shown that hybrid immunity correlates with higher level of antibodies, improved protection against severe COVID-19 disease and potentially longer duration of protection, all of which could have impacted time of vaccination and our estimates [31]. 

This study has several strengths compared with published research. It is one of a limited number assessing diverse PROs associated with COVID-19 at community pharmacies. As such, it adds to existing evidence to contribute a holistic picture of humanistic outcomes associated with symptomatic COVID-19, assessed directly from a patient’s perspective. From an internal validity perspective, the study enrolled patients within days from testing positive and prospectively collected survey-based data shortly after infection, potentially minimizing recall bias. Moreover, the study leveraged widely used validated PRO instruments (EQ-5D-5L, WPAI, PROMIS Fatigue) and a questionnaire capturing a comprehensive symptoms list aligned to CDC research. With asymptomatic infections excluded by design, our estimates can be interpreted exclusively as related to symptomatic disease. Further, both model-based and matching analyses yielded consistent results. Finally, with all study activities carried out virtually, this study piloted an innovative approach to agile and digitally enabled research during a pandemic.

The study is subject to several limitations. As previously described [5], all data collected were self-reported, subject to missingness, errors, recall bias, social desirability bias and selection bias associated with survey drop-off. Out of 643 participants, 26.7% were lost to follow-up at Week 4, possibly due to survey fatigue. Such drop-off rate should be interpreted in the context of participants being asked not to skip surveys. Such a strict requirement allowed for a clean assessment of changes in outcomes prevalence over time, but at the cost of attrition. Other limitations include over-representation of females, the relatively healthy baseline status of the population, exclusion of pediatrics, the fact that the study did not assess symptom severity, immunity levels, and that WPAI analyses were impacted by smaller sample size. Moreover, despite adjusting for several covariates in the model, risk of residual confounding may remain. These findings may not be generalizable to other settings, prior or future variants, other countries, time periods and populations that were excluded. Finally, this study did not explore patient views, perceptions, and barriers to prevention.

While this study contributes to addressing knowledge gaps on symptomatology and PROs associated with COVID-19, symptoms are numerous and heterogeneous, and characterization of the acute infection continues to evolve. To our knowledge, this is the first study that provides estimates of the impact of bivalent BA.4/5 BNT162b2 on PROs in the context of high seroprevalence. As such, it adds novel insights and, when taken together with the prior monovalent BNT162b2 study [4,5] and similar studies [1,2,3], it solidifies evidence indicating that the effectiveness of BA.4/5 BNT162b2 on COVID-19 disease could translate to extra benefits of reduction in the frequency and burden of symptoms, supporting faster recovery and return to work. Future studies, especially evaluating variant-updated COVID-19 vaccine formulations, could corroborate these findings with different designs (for example, with a test-negative control), use of COVID-19 specific validated instruments, and assess these outcomes in subgroups defined by socio-demographic characteristics and antiviral treatment history. 

## 6. Conclusions

This study shows that COVID-19 adversely affects patients’ wellbeing, productivity and activity levels. Additionally, the results show the benefits of bivalent BA.4/5 BNT162b2 vaccine in alleviating symptoms and enhancing work productivity after acute SARS-CoV2 infection. These findings, taken together with existing evidence, show a consistent additive benefit of BNT162b2 beyond traditional endpoints, even with a new formulation and evolving sub-lineages. 

## Figures and Tables

**Table 1:** Baseline characteristics stratified by vaccination status.

	All	BNT162b2	Unvaccinated	*p* ^a^
**Total n (%)**	643 (100.0)	316 (49.1)	327 (50.9)	
**Age, years, mean (SD)**	46.5 (15.9)	50.8 (16.0)	42.3 (14.7)	<0.001
**Age group, n (%)**				<0.001
18–29	109 (17.0)	33 (10.4)	76 (23.2)	
30–49	257 (40.0)	111 (35.1)	146 (44.6)	
50–64	167 (26.0)	93 (29.4)	74 (22.6)	
≥65–74	110 (17.0)	79 (25.0)	31 (9.5)	
**Gender, n (%)**				0.185
Female	452 (70.3)	212 (67.1)	240 (73.4)	
Male	185 (28.8)	101 (32.0)	84 (25.7)	
Unknown	6 (0.9)	3 (1.0)	3 (0.9)	
**Race/Ethnicity, n (%)**				<0.001
White or Caucasian (not Hispanic or Latino)	374 (58.2)	204 (64.6)	170 (52.0)	
Black or African American	57 (8.9)	19 (6.0)	38 (11.6)	
Hispanic	99 (15.4)	33 (10.4)	66 (20.2)	
Asian	63 (9.8)	36 (11.4)	27 (8.3)	
Patient Refused/Other	50 (7.7)	24 (7.6)	26 (8.0)	
**US Geographic Region, n (%)**				0.013
Northeast	88 (13.7)	51 (16.1)	37 (11.3)	
South	260 (40.4)	108 (34.2)	152 (46.5)	
Midwest	141 (21.9)	76 (24.1)	65 (19.9)	
West	154 (24.0)	81 (25.6)	73 (22.3)	
**Social vulnerability index, Mean (SD) ^b^**	0.45 (0.2)	0.39 (0.2)	0.50 (0.2)	0.000
**Previously Tested Positive, n (%)**				0.051
No	338 (52.6)	177 (56.0)	161 (49.2)	
Yes	268 (41.7)	119 (37.7)	149 (45.6)	
Missing	37 (5.8)	20 (6.3)	17 (5.3)	
**Number of comorbidities, Mean (SD)**	0.40 (0.8)	0.47 (0.8)	0.34 (0.8)	0.043
**At least 1 comorbidity, n (%)**	165 (25.7)	96 (30.4)	69 (21.1)	0.007
Asthma or Chronic Lung Disease, n (%)	33 (5.1)	20 (6.3)	13 (4.0)	0.176
Cirrhosis of the liver, n (%)	2 (0.3)	0 (0.0)	2 (0.6)	0.499
Immunocompromised Conditions or Weakened Immune System ^c^, n (%)	2 (0.3)	1 (0.3)	1 (0.3)	1.000
Diabetes, n (%)	39 (6.1)	23 (7.3)	16 (4.9)	0.205
Heart Conditions or Hypertension, n (%)	104 (16.2)	60 (19.0)	44 (13.5)	0.057
Overweight or obesity, n (%)	80 (12.4)	44 (13.9)	36 (11.0)	0.263
**Paxlovid prescription, n (%)**				0.000
No	493 (76.7)	222 (70.3)	271 (82.9)	
Yes	148 (23.0)	92 (29.1)	56 (17.1)	
Missing	2 (0.3)	2 (0.6)	0 (0.0)	

CMS: Centers for Medicare and Medicaid Services; SD: Standard Deviation. ^a^ *p* value refers to the comparison between BNT162b2 and Unvaccinated. ^b^ SVI ranges from 0 to 1. A community with higher value is more socially vulnerable. ^c^ Immunocompromised conditions includes compromised immune system (such as from immune-compromising drugs, solid organ or blood stem cell transplant, HIV, or other conditions), conditions that result in a weakened immune system, including kidney failure or end stage renal disease.

**Table 2:** Trajectory of acute COVID-19 symptoms at time of testing, Week 1, Week 2 and Week 4.

	All	BNT162b2	Unvaccinated	*p* ^a^	*p* ^b^
**Index day (time of testing)**					
n	643 (100.0)	316 (49.1)	327 (50.9)		
Mean number of symptoms (SD)	5.3 (2.3)	5.0 (2.3)	5.7 (2.2)	0.001	0.002
Median (Q1, Q3)	5 (4.0, 7.0)	5 (3.0, 7.0)	6 (4.0, 7.0)	0.000	
**Number of ARI symptoms, n (%)**				0.007	
1–2	75 (11.7)	45 (14.2)	30 (9.2)		
3–5	271 (42.1)	146 (46.2)	125 (38.2)		
6+	297.2 (46.2)	125 (39.5)	172 (52.6)		
**Systemic symptoms, n (%)**	574 (89.3)	273 (86.4)	301 (92.0)	0.021	0.037
Fever	268 (41.7)	119 (37.7)	149 (45.6)	0.042	0.037
Chills	332 (51.6)	140 (44.3)	192 (58.7)	0.000	0.201
Muscle or Body Aches	297 (46.2)	125 (39.6)	172 (52.6)	0.001	0.030
Headache	417 (64.9)	191 (60.4)	226 (69.1)	0.021	0.010
Fatigue	424 (65.9)	202 (63.9)	222 (67.9)	0.289	0.164
**Respiratory symptoms, n (%)**	627 (97.5)	310 (98.1)	317 (96.9)	0.345	0.359
Shortness of Breath or Difficulty Breathing	101 (15.7)	48 (15.2)	53 (16.2)	0.723	0.568
Cough	491 (76.4)	237 (75.0)	254 (77.7)	0.425	0.401
Sore Throat	390 (60.7)	191 (60.4)	199 (60.9)	0.915	0.571
New/Recent Loss of Taste or Smell	89 (13.8)	37 (11.7)	52 (15.9)	0.124	0.185
Congestion or Runny Nose	525 (81.6)	258 (81.6)	267 (81.7)	0.999	0.908
**GI symptoms, n (%)**	92 (14.3)	37 (11.7)	55 (16.8)	0.064	0.500
Nausea or Vomiting	25 (3.9)	10 (3.2)	15 (4.6)	0.351	0.670
Diarrhea	80 (12.4)	33 (10.4)	47 (14.4)	0.131	0.443
**Week 1**					
n	566	285 (50.4)	281 (49.6)		
Mean number of symptoms (SD)	2.6 (1.8)	2.5 (1.6)	2.7 (1.9)	0.129	0.368
Median, Q1–Q3	2 (1.0, 3.0)	2 (1.0, 3.0)	2 (1.0, 4.0)	0.242	
**Number of ARI symptoms**				0.158	
1–2	340 (60.0)	179 (62.8)	161 (57.1)		
3–5	189 (33.3)	94 (33.0)	95 (33.7)		
6+	37 (6.5)	12 (4.3)	25 (8.9)		
Missing	1 (0.2)	0 (0.0)	1 (0.4)		
**Systemic symptoms, n (%)**	332 (58.6)	156 (54.7)	176 (62.4)	0.064	0.127
Fever	15 (2.7)	3 (1.1)	12 (4.3)	0.017	0.037
Chills	21 (3.7)	10 (3.5)	11 (3.9)	0.798	0.902
Muscle or Body Aches	87 (15.4)	34 (11.9)	53 (18.9)	0.022	0.072
Headache	129 (22.8)	50 (17.5)	79 (28.1)	0.003	0.034
Fatigue	286 (50.5)	139 (48.8)	147 (52.3)	0.400	0.497
**Respiratory symptoms, n (%)**	418 (73.7)	212 (74.4)	206 (73.0)	0.718	0.798
Shortness of Breath or Difficulty Breathing	95 (16.8)	44 (15.4)	51 (18.1)	0.388	0.670
Cough	284 (50.2)	141 (49.5)	143 (50.9)	0.736	0.644
Sore Throat	79 (14.0)	39 (13.7)	40 (14.2)	0.850	0.951
New/Recent Loss of Taste or Smell	76 (13.4)	37 (13.0)	39 (13.9)	0.754	0.907
Congestion or Runny Nose	247 (43.6)	132 (46.3)	115 (40.9)	0.196	0.298
**GI symptoms, n (%)**	46 (8.1)	24 (8.4)	22 (7.8)	0.787	0.471
Nausea or Vomiting	3 (0.5)	1 (0.4)	2 (0.7)	0.554	0.689
Diarrhea	43 (7.6)	23 (8.1)	20 (7.1)	0.669	0.561
**Week 2**					
n	530	269 (50.8)	261 (49.2)		
Mean number of symptoms (SD)	1.9 (1.3)	1.9 (1.3)	2.0 (1.4)	0.133	0.465
Median, Q1–Q3	1 (1.0, 3.0)	1 (1.0, 2.0)	1 (1.0, 3.0)	0.270	
Number of ARI symptoms				0.284	
1–2	397 (74.9)	209 (77.7)	188 (72.0)		
3–5	120 (22.6)	55 (20.4)	65 (24.9)		
6–8	13 (2.5)	5 (1.9)	8 (3.1)		
**Systemic symptoms, n (%)**	254 (47.9)	125 (46.5)	129 (49.4)	0.496	0.638
Fever	7 (1.3)	2 (0.7)	5 (1.9)	0.237	0.289
Chills	12 (2.3)	5 (1.9)	7 (2.7)	0.524	0.613
Muscle or Body Aches	80 (15.1)	36 (13.4)	44 (16.9)	0.264	0.391
Headache	79 (14.9)	33 (12.3)	46 (17.6)	0.083	0.224
Fatigue	218 (41.1)	108 (40.1)	110 (42.1)	0.640	0.780
**Respiratory symptoms, n (%)**	271 (51.1)	134 (49.8)	137 (52.5)	0.538	0.299
Shortness of Breath or Difficulty Breathing	51 (9.6)	22 (8.2)	29 (11.1)	0.252	0.474
Cough	184 (34.7)	93 (34.6)	91 (34.9)	0.943	0.731
Sore Throat	44 (8.3)	17 (6.3)	27 (10.3)	0.093	0.100
New/Recent Loss of Taste or Smell	29 (5.5)	17 (6.3)	12 (4.6)	0.384	0.310
Congestion or Runny Nose	125 (23.6)	64 (23.8)	61 (23.4)	0.909	0.979
**GI symptoms, n (%)**	31 (5.9)	14 (5.2)	17 (6.5)	0.521	0.860
Nausea or Vomiting	3 (0.6)	2 (0.7)	1 (0.4)	0.580	0.476
Diarrhea	29 (5.5)	13 (4.8)	16 (6.1)	0.511	0.655
**Week 4**					
n	505	260 (51.5)	245 (48.5)		
Mean number of symptoms (SD)	0.9 (1.3)	0.7 (1.2)	1.1 (1.4)	0.002	0.033
Median, Q1–Q3	0 (0.0, 1.0)	0 (0.0, 1.0)	1 (0.0, 2.0)	0.000	
Number of ARI symptoms				0.009	
0	272 (53.8)	159 (61.2)	113 (45.9)		
1–2	178 (35.2)	80 (30.8)	98 (39.8)		
3–5	46 (9.1)	17 (6.5)	29 (11.8)		
6–8	9 (1.8)	4 (1.5)	5 (2.0)		
Missing	1 (0.0)	0 (0.0)	1 (0.0)		
**Systemic symptoms, n (%)**	167 (33.0)	67 (25.8)	100 (40.7)	0.000	0.005
Fever	5 (1.0)	2 (0.8)	3 (1.2)	0.606	0.658
Chills	6 (1.2)	3 (1.2)	3 (1.2)	0.942	0.992
Muscle or Body Aches	42 (8.3)	17 (6.5)	25 (10.2)	0.136	0.229
Headache	67 (13.3)	30 (11.5)	37 (15.1)	0.238	0.576
Fatigue	123 (24.4)	46 (17.7)	77 (31.4)	0.000	0.003
**Respiratory symptoms, n (%)**	138 (27.3)	61 (23.5)	77 (31.3)	0.048	0.027
Shortness of Breath or Difficulty Breathing	50 (9.9)	22 (8.5)	28 (11.4)	0.265	0.516
Cough	93 (18.4)	43 (16.5)	50 (20.4)	0.262	0.201
Sore Throat	21 (4.2)	6 (2.3)	15 (6.1)	0.032	0.057
New/Recent Loss of Taste or Smell	18 (3.6)	8 (3.1)	10 (4.1)	0.543	0.691
Congestion or Runny Nose	N/A				
**GI symptoms, n (%)**	30 (5.9)	12 (4.6)	18 (7.3)	0.198	0.409
Nausea or Vomiting	18 (3.6)	7 (2.7)	11 (4.5)	0.276	0.521
Diarrhea	15 (3.0)	5 (1.9)	10 (4.1)	0.153	0.212

^a^ *p* values of *t*-test for number of symptoms, Chi-square tests or Fisher’s exact tests when any one cell has an expected frequency less than 5 for individual symptoms and number of symptom category comparing the BNT162b2 cohort and the unvaccinated cohort. ^b^ Model-based *p* value.

## Data Availability

Aggregated data that support the findings of this study are available upon reasonable request from the corresponding author (M.D.F.), subject to review. These data are not publicly available due to them containing information that could compromise research participant privacy/consent.

## Supplementary Materials

The following supporting information can be downloaded at: https://www.mdpi.com/article/10.3390/vaccines11111669/s1, Table S1: Patient characteristics by enrollment status. Table S2: Patient characteristics by vaccination status after matching. Table S3: Trajectory of acute COVID-19 symptoms at time of testing, Week 1, Week 2 and Week 4 after matching. Table S4: Summary of observed EQ-5D-5L, PROMIS Fatigue and WPAI-GH scores and their changes from pre-COVID-19 baseline by assessment time and vaccination status. Table S5: Least-square mean estimates of EQ-5D-5L, PROMIS Fatigue and WPAI-GH scores and their changes from pre-COVID-19 baseline by assessment time and vaccination status. Table S6: Mixed models for repeated measurements of EQ-5D-5L and WPAI-GH scores. Table S7: EQ-5D-5L and WPAI-GH scores post-matching. Figure S1: SARS-CoV-2 variant proportions in the US. Figure S2: Prevalence of acute COVID-19 symptoms by vaccination status. Figure S3: Prevalence of acute COVID-19 symptoms over time by category.
