# Supplementary material for: Impact of Bivalent BA.4/5 BNT162b2 COVID-19 Vaccine on Acute Symptoms, Quality of Life, Work Productivity and Activity Levels among Symptomatic US Adults Testing Positive for SARS-CoV-2 at a National Retail Pharmacy
Source: Vaccines (Basel). 2023 Oct 31;11(11):1669. doi: 10.3390/vaccines11111669 (PMC10675533; doi:10.3390/vaccines11111669)

Impact of bivalent BA.4/5 BNT162b2 COVID-19 vaccine on acute symptoms, quality of life, work productivity and activity levels among symptomatic US adults testing positive for SARS-CoV-2 at a national retail pharmacy

Supplementary Material

Table S1 Patient Characteristics by Enrollment Status

Table S2 Patient Characteristics by Vaccination Status after matching

Table S3: Trajectory of acute COVID-19 symptoms at time of testing, Week 1, Week 2 and Week 4 after matching

Table S4 Summary of Observed EQ-5D-5L, PROMIS Fatigue and WPAI-GH Scores and Their Changes from Pre-COVID-19 Baseline by Assessment Time and Vaccination Status.

Table S5 Least-Square Mean Estimates of EQ-5D-5L, PROMIS Fatigue and WPAI-GH Scores and Their Changes from Pre-COVID-19 Baseline by Assessment Time and Vaccination Status

Table S6 Mixed Models for Repeated Measurements of EQ-5D-5L and WPAI-GH scores

Table S7. EQ-5D-5L and WPAI-GH Scores post-matching

Figure S1 SARS-CoV-2 Variants proportions in the US

Figure S2 Prevalence of acute COVID-19 symptoms by vaccination status

Figure S3 Prevalence of acute COVID-19 symptoms over time by category

Table S1 Patient Characteristics by Enrollment Status

	All	Included	Excluded	*P* [a]
Total, n (%)	21,027	643	20,384	
Age, years				
Mean, SD	45.3 (16.9)	46.5 (15.9)	45.2 (17.0)	0.064
Age group, n (%)				0.001
18-29	4,749 (22.6)	109 (17.0)	4,640 (22.8)	
30-49	7,661 (36.4)	257 (40.0)	7,404 (36.3)	
50-64	5,276 (25.1)	167 (26.0)	5,109 (25.1)	
65-74	2,371 (11.3)	89 (13.8)	2,282 (11.2)	
≥75	970 (4.6)	21 (3.2)	949 (4.7)	
Gender, n (%)				<0.001
Female	12,441 (59.2)	452 (70.3)	11,989 (58.8)	
Male	8,428 (40.1)	185 (28.8)	8,243 (40.4)	
Unknown	158 (0.7)	6 (0.9)	152 (0.8)	
Race / Ethnicity, n (%)				<0.001
White or Caucasian (not Hispanic or Latino)	8,626 (41.0)	374 (58.2)	8,252 (40.5)	
Black or African American	3,014 (14.3)	57 (8.9)	2,957 (14.5)	
Hispanic	5,094 (24.2)	99 (15.4)	4,995 (24.5)	
Asian	2,462 (11.7)	63 (9.8)	2,399 (11.8)	
Patient Refused	1,051 (5.0)	22 (3.4)	1,029 (5.1)	
Other	777 (3.7)	28 (4.3)	749 (3.7)	
Missing	3 (0.0)	0 (0.0)	3 (0.0)	
US Geographic Region, n (%)				0.037
Northeast	2,493 (11.9)	88 (13.7)	2,405 (11.8)	
South	9,517 (45.3)	260 (40.4)	9,257 (45.4)	
Midwest	3,847 (18.3)	141 (21.9)	3,706 (18.2)	
West	5,168 (24.6)	154 (24.0)	5,014 (24.6)	
Missing	2 (0.0)	0 (0.0)	2 (0.0)	
CMS Geographic Region (n, %)				0.002
Region 1: ME, NH, VT, MA, CT, RI	1,088 (5.2)	43 (6.7)	1,045 (5.1)	
Region 2: NY, NJ, PR, VI	684 (3.3)	21 (3.3)	663 (3.3)	
Region 3: PA, DE, MD, DC, WV, VA	1,611 (7.7)	52 (8.1)	1,559 (7.7)	
Region 4: KY, TN, NC, SC, GA, MS, AL, FL	4,742 (22.6)	146 (22.7)	4,596 (22.5)	

	All	Included	Excluded	*P* [a]
Region 5: MN, WI, IL, MI, IN, OH	3,507 (16.7)	132 (20.5)	3,375 (16.6)	
Region 6: NM, OK, AR, TX, LA	3,967 (18.9)	88 (13.7)	3,879 (19.0)	
Region 7: NE, IA, KS, MO	336 (1.6)	9 (1.4)	327 (1.6)	
Region 8: MT, ND, SD, WY, UT, CO	60 (0.3)	5 (0.8)	55 (0.3)	
Region 9: CA, NV, AZ, GU	4,958 (23.6)	142 (22.1)	4,816 (23.6)	
Region 10: AK, WA, OR, ID	73 (0.4)	5 (0.8)	68 (0.3)	
Missing	1 (0.0	0 (0.0)	1 (0.0)	
Social vulnerability index, Mean (SD)[b]	0.48 (0.2)	0.45 (0.2)	0.48 (0.2)	<0.001
Previously Tested Positive, n (%)				0.532
No	10,607 (50.4)	338 (52.6)	10,269 (50.4)	
Yes	8,844 (42.1)	268 (41.7)	8,576 (42.1)	
Missing	1,576 (7.5)	37 (5.8)	1,539 (7.6)	
Number of comorbidities, Mean (SD)	0.30 (0.7)	0.40 (0.8)	0.30 (0.7)	0.001
At least 1 comorbidity, n (%)	4,138 (19.7)	165 (25.7)	3,973 (19.5)	<0.001
Self-Reported Comorbidity, n (%)				
Asthma or Chronic Lung Disease	738 (3.5)	33 (5.1)	705 (3.5)	0.023
Cirrhosis of the liver	51 (0.2)	2 (0.3)	49 (0.2)	0.720
Immunocompromised Conditions or Weakened Immune System[c]	106 (0.5)	2 (0.3)	104 (0.5)	0.483
Diabetes	1,277 (6.1)	39 (6.1)	1,238 (6.1)	0.993
Heart Conditions or Hypertension	2,783 (13.2)	104 (16.2)	2,679 (13.1)	0.026
Overweight or obesity	1,625 (7.7)	80 (12.4)	1,545 (7.6)	<0.001
**Index day[d] acute COVID-19 symptoms**				
Number of symptoms, Mean, SD	5.30 (2.40)	5.30 (2.30)	5.30 (2.4)	0.484
Fever	9,033 (43.0)	268 (41.7)	8,765 (43.0)	0.506
Chills	3,225 (15.3)	101 (15.7)	3,124 (15.3)	0.791
Muscle or Body Aches	16,798 (79.9)	491 (76.4)	16,307 (80.0)	0.023

	All	Included	Excluded	*P* [a]
Headache	11,058 (52.6)	332 (51.6)	10,726 (52.6)	0.622
Fatigue	1,019 (4.9)	25 (3.9)	994 (4.9)	0.251
Shortness of Breath or Difficulty Breathing	9,956 (47.3)	297 (46.2)	9,659 (47.4)	0.550
Cough	11,935 (56.8)	390 (60.7)	11,545 (56.6)	0.043
Sore Throat	2,879 (13.7)	89 (13.8)	2,790 (13.7)	0.911
New/Recent Loss of Taste or Smell	12,441 (59.2)	424 (65.9)	12,017 (59.0)	0.000
Congestion or Runny Nose	13,100 (62.3)	417 (64.9)	12,683 (62.2)	0.175
Nausea or Vomiting	16,398 (78.0)	525 (81.6)	15,873 (77.9)	0.023
Diarrhea	3,230 (15.4)	80 (12.4)	3,150 (15.5)	0.037

CMS: Centers for Medicare and Medicaid Services; SD: Standard Deviation
[a] *P* value refers to the comparison between Included and Excluded.
[b] SVI ranges from 0 to 1. A community with higher value is more socially vulnerable.
[c] Immunocompromised conditions includes compromised immune system (such as from immuno-compromising drugs, solid organ or blood stem cell transplant, HIV, or other conditions), conditions that result in a weakened immune system, including kidney failure or end stage renal disease
[d] COVID-19 test nasal swab day

Table S2 Patient Characteristics by Vaccination Status after matching

	All	BNT162b2	Unvaccinated	*P* [a]
Total, n (%)	643	316 (49.1)	327 (50.9)	
Age, years				
Mean, SD	46.5 (15.9)	46.6 (15.9)	46.4 (15.9)	0.917
Age group, n (%)				0.776
18-29	109 (17.0)	54 (17.0)	56 (17.0)	
30-49	257 (40.0)	126 (40.0)	131 (40.0)	
50-64	167 (26.0)	82 (26.0)	85 (26.0)	
65-74	89 (13.8)	44 (13.8)	45 (13.8)	
≥75	21 (3.2)	10 (3.2)	11 (3.2)	
Gender, n (%)				1.000
Female	458 (71.1)	221 (70.0)	236 (72.2)	
Male	178 (27.7)	91 (28.6)	88 (26.8)	
Unknown	7 (1.2)	4 (1.3)	3.1 (1.0)	
Race / Ethnicity, n (%)				1.000
White or Caucasian (not Hispanic or Latino)	374 (58.2)	184 (58.2)	190 (58.2)	
Black or African American	57 (8.9)	28 (8.9)	29 (8.9)	
Hispanic	99 (15.4)	49 (15.4)	50 (15.4)	
Asian	63 (9.8)	31 (9.8)	32 (9.8)	
Patient Refused	22 (3.4)	11 (3.4)	11 (3.4)	
Other	28 (4.3)	14 (4.3)	14 (4.3)	
US Geographic Region, n (%)				0.114
Northeast	88 (13.7)	43 (13.7)	45 (13.7)	
South	260 (40.4)	128 (40.4)	132 (40.4)	
Midwest	141 (21.9)	69 (21.9)	72 (21.9)	
West	154 (24.0)	76 (24.0)	79 (24.0)	
CMS Geographic Region (n, %)				0.090
Region 1: ME, NH, VT, MA, CT, RI	43 (6.8)	23 (7.2)	21 (6.4)	
Region 2: NY, NJ, PR, VI	23 (3.6)	10 (3.3)	13 (3.9)	
Region 3: PA, DE, MD, DC, WV, VA	57 (8.8)	34 (10.9)	22 (6.7)	
Region 4: KY, TN, NC, SC, GA, MS, AL, FL	143 (22.3)	69 (21.9)	74 (22.7)	
Region 5: MN, WI, IL, MI, IN, OH	132 (20.5)	64 (20.3)	68 (20.7)	
Region 6: NM, OK, AR, TX, LA	83 (12.8)	35 (11.0)	48 (14.6)	

	All	BNT162b2	Unvaccinated	P [a]
Region 7: NE, IA, KS, MO	9 (1.4)	5 (1.6)	4 (1.2)	
Region 8: MT, ND, SD, WY, UT, CO	5 (0.7)	4 (1.2)	1 (0.2)	
Region 9: CA, NV, AZ, GU	143 (22.2)	66 (20.7)	77 (23.6)	
Region 10: AK, WA, OR, ID	6 (0.9)	6 (1.9)	0 (0.0)	
Social vulnerability index, Mean (SD)[b]	0.44 (0.2)	0.44 (0.2)	0.45 (0.2)	0.801
Previously Tested Positive, n (%)				0.090
No	328 (54.6)	170 (58.1)	158 (51.2)	
Yes	273 (45.4)	123 (41.9)	151 (48.8)	
Missing	37	20	17	
Number of comorbidities, Mean (SD)	0.40 (0.8)	0.40 (0.8)	0.40 (0.8)	0.770
At least 1 comorbidity, n (%)	165 (25.7)	81 (25.7)	84 (25.7)	1.000
Self-Reported Comorbidity, n (%)				
Asthma or Chronic Lung Disease	37 (5.8)	20 (6.4)	17 (5.2)	0.506
Cirrhosis of the liver	2 (0.4)	0 (0.0)	2 (0.7)	0.133
Immunocompromised Conditions or Weakened Immune System[c]	2 (0.3)	1 (0.3)	1 (0.2)	0.849
Diabetes	39 (6.0)	21 (6.5)	18 (5.5)	0.596
Heart Conditions or Hypertension	100 (15.5)	44 (14.0)	56 (17.0)	0.292
Overweight or obesity	80 (12.4)	39 (12.2)	41 (12.6)	0.878
Smoking	25 (3.8)	11 (3.4)	14 (4.2)	0.572
Paxlovid prescription, n (%)				1.000
No	495 (77.0)	243 (77.0)	252 (77.0)	
Yes	148 (23.0)	73 (23.0)	75 (23.0)	
Missing	2	2	0	

CMS: Centers for Medicare and Medicaid Services; SD: Standard Deviation
[a] *P* value refers to the comparison between BNT162b2 and Unvaccinated.
[b] SVI ranges from 0 to 1. A community with higher value is more socially vulnerable.
[c] Immunocompromised conditions includes compromised immune system (such as from immuno-compromising drugs, solid organ or blood stem cell transplant, HIV, or other conditions), conditions that result in a weakened immune system, including cancer treatment, and kidney failure or end stage renal disease

Table S3 Trajectory of acute COVID-19 symptoms at time of testing, Week 1, Week 2 and Week 4 after matching

	All	BNT162b2	Unvaccinated	*P* [a]
**Index day (time of testing)**				
n	643	316 (49.1)	327 (50.9)	
Mean number of symptoms (SD)	5.3 (2.3)	5.1 (2.4)	5.5 (2.2)	0.008
Median (Q1, Q3)	5 (3.0, 7.0)	5 (3.0, 7.0)	6 (4.0, 7.0)	
Min, max	1, 12	1, 12	1, 11	
Missing	0	0	0	
Number of ARI symptoms, n (%)				0.022
<3	79 (12.4)	46 (14.6)	33 (10.1)	
3-5	273 (42.5)	143 (45.2)	130 (39.8)	
6-8	235 (36.5)	97 (30.8)	137 (42.0)	
9+	56 (8.7)	30 (9.4)	27 (8.1)	
Systemic symptoms, n (%)	569 (88.4)	271 (85.7)	298 (91.1)	0.032
Fever	263 (40.8)	117 (37.0)	146 (44.6)	0.050
Chills	327 (50.8)	142 (44.8)	185 (56.6)	0.003
Muscle or Body Aches	285 (44.2)	120 (37.8)	165 (50.4)	0.002
Headache	417 (64.8)	195 (61.5)	222 (68.0)	0.085
Fatigue	417 (64.9)	201 (63.7)	216 (66.1)	0.523
Respiratory symptoms, n (%)	629 (97.8)	311 (98.3)	318 (97.3)	0.375
Shortness of Breath or Difficulty Breathing	100 (15.5)	50 (15.7)	50 (15.4)	0.910
Cough	493 (76.7)	237 (74.9)	256 (78.4)	0.304
Sore Throat	393 (61.1)	196 (61.9)	197 (60.3)	0.685
New/Recent Loss of Taste or Smell	83 (12.9)	38 (12.1)	45 (13.7)	0.544
Congestion or Runny Nose	531 (82.5)	260 (82.3)	271 (82.8)	0.862
GI symptoms, n (%)	88 (13.6)	37 (11.6)	51 (15.6)	0.143
Nausea or Vomiting	23 (3.6)	10 (3.3)	12 (3.8)	0.724
Diarrhea	76 (11.8)	33 (10.3)	43 (13.2)	0.264
**Week 1**				
n	566	285 (50.4)	281 (49.6)	
Mean number of symptoms (SD)	2.5 (1.7)	2.5 (1.7)	2.6 (1.8)	0.365
Median, Q1-Q3	2 (1.0, 3.0)	2 (1.0, 3.0)	2 (1.0, 3.0)	
Min, max	1, 10	1, 10	1, 9	
Missing	1	0	1	
Number of ARI symptoms				0.466
<3	350 (61.7)	183 (64.0)	167 (59.3)	

3-5	184 (32.4)	90 (31.5)	94 (33.4)	
6-8	24 (4.3)	9 (3.2)	15 (5.4)	
9+	9 (1.6)	4 (1.3)	5 (1.8)	
Missing	1 (0.1)	0 (0.0)	1 (0.2)	
Systemic symptoms, n (%)	329 (58.1)	155 (54.5)	174 (61.7)	0.083
Fever	15 (2.6)	3 (1.2)	12 (4.1)	0.016
Chills	20 (3.5)	9 (3.3)	11 (3.8)	0.607
Muscle or Body Aches	83 (14.7)	33 (11.7)	50 (17.7)	0.034
Headache	123 (21.8)	52 (18.4)	71 (25.2)	0.042
Fatigue	281 (49.6)	136 (47.7)	145 (51.6)	0.337
Respiratory symptoms, n (%)	415 (73.2)	209 (73.5)	206 (73.0)	0.720
Shortness of Breath or Difficulty Breathing	89 (15.7)	45 (15.9)	42 (15.4)	0.820
Cough	276 (48.7)	137 (48.0)	139 (49.4)	0.816
Sore Throat	79 (13.9)	42 (14.8)	37 (13.0)	0.591
New/Recent Loss of Taste or Smell	73 (12.9)	36 (12.7)	37 (13.2)	0.741
Congestion or Runny Nose	249 (43.9)	134 (47.0)	115 (40.7)	0.130
GI symptoms, n (%)	41 (7.3)	23 (7.9)	19 (6.7)	0.643
Nausea or Vomiting	2 (0.4)	1 (0.3)	1 (0.5)	0.741
Diarrhea	39 (6.9)	22 (7.6)	18 (6.3)	0.583
**Week 2**				
n	530	269 (50.8)	261 (49.2)	
Mean number of symptoms (SD)	1.9 (1.3)	1.9 (1.3)	2.0 (1.4)	0.182
Median, Q1-Q3	1 (1.0, 2.0)	1 (1.0, 2.0)	1 (1.0, 3.0)	
Min, max	1, 8	1, 8	1, 8	
Missing	0	0	0	
Number of ARI symptoms				0.242
<3	401 (75.7)	210 (78.2)	191 (73.1)	
3-5	117 (22.0)	54 (19.9)	63 (24.2)	
6-8	12 (2.3)	5 (1.9)	7 (2.7)	
Systemic symptoms, n (%)	254 (48.0)	126 (47)	128 (48.9)	0.508
Fever	7 (1.4)	3 (0.9)	5 (1.8)	0.405
Chills	13 (2.4)	6 (2.1)	7 (2.7)	0.663
Muscle or Body Aches	72 (13.7)	34 (12.5)	39 (14.8)	0.340
Headache	80 (15.2)	34 (12.6)	47 (17.8)	0.084
Fatigue	218 (41.2)	110 (40.7)	109 (41.8)	0.675
Respiratory symptoms, n (%)	270 (50.9)	129 (47.8)	141 (54.1)	0.139
Shortness of Breath or Difficulty Breathing	48 (9.1)	21 (7.9)	27 (10.3)	0.367
Cough	181 (34.1)	91 (33.8)	90 (34.4)	0.853
Sore Throat	43 (8.2)	16 (5.9)	28 (10.5)	0.039

New/Recent Loss of Taste or Smell	29 (5.4)	16 (5.9)	13 (4.8)	0.870
Congestion or Runny Nose	128 (24.2)	65 (24.1)	64 (24.4)	0.822
GI symptoms, n (%)	27 (5.1)	13 (4.9)	14 (5.3)	0.855
Nausea or Vomiting	3 (0.6)	3 (0.9)	1 (0.3)	0.302
Diarrhea	25 (4.7)	12 (4.5)	13 (5.0)	0.784
**Week 4**				
n	505	260 (51.5)	245 (48.5)	
Mean number of symptoms (SD)	0.8 (1.3)	0.7 (1.2)	1.2 (1.0)	0.004
Median, Q1-Q3	0 (0.0, 1.0)	0 (0.0, 1.0)	1 (0.0, 2.0)	
Min, max	0, 8	0, 7	0, 8	
Missing	1	0	1	
Number of ARI symptoms				0.005
0	275 (54.4)	161 (61.9)	114 (46.5)	
1-2	182 (35.9)	81 (31.0)	101 (41.0)	
3-5	42 (8.3)	15 (5.6)	27 (11.0)	
6-8	7 (1.4)	4 (1.5)	3 (1.3)	
Missing	1 (0.1)	0 (0.0)	1 (0.2)	
Systemic symptoms, n (%)	161 (31.9)	66 (25.5)	95 (38.6)	0.001
Fever	7 (1.4)	3 (1.3)	4 (1.4)	0.889
Chills	7 (1.4)	4 (1.7)	3 (1.1)	0.672
Muscle or Body Aches	39 (7.8)	15 (5.8)	24 (9.7)	0.036
Headache	64 (12.6)	29 (11.1)	35 (14.2)	0.324
Fatigue	115 (22.8)	44 (17.1)	71 (28.8)	0.001
Respiratory symptoms, n (%)	129 (25.5)	57 (22.1)	72 (29.1)	0.042
Shortness of Breath or Difficulty Breathing	48 (9.5)	22 (8.6)	26 (10.4)	0.415
Cough	85 (16.8)	39 (15.0)	46 (18.8)	0.162
Sore Throat	19 (3.7)	5 (2.0)	14 (5.5)	0.042
New/Recent Loss of Taste or Smell	14 (2.8)	6 (2.4)	8 (3.3)	0.405
Congestion or Runny Nose	N/A			
GI symptoms, n (%)	27 (5.3)	12 (4.7)	15 (6.0)	0.537
Nausea or Vomiting	14 (2.8)	5 (2.1)	9 (3.5)	0.348
Diarrhea	15 (2.9)	7 (2.6)	8 (3.2)	0.694

[a] *P* values refers to the comparison between BNT162b2 and Unvaccinated, after matching by time point on age, race/ethnicity, region, SVI category, Paxlovid use, ≥1 comorbidity.

Table S4 Summary of Observed EQ-5D-5L, PROMIS Fatigue and WPAI-GH Scores a and Their Changes from Pre-COVID-19 Baseline by Assessment Time and Vaccination Status.

	**BNT162b2**						**Unvaccinated**						**Difference in Change from Baseline Between Cohorts**		
	**Score**		**Change from Baseline [b]**				**Score**		**Change from Baseline**						
	**n**	**Mean (SD)**	**n**	**Mean (SD)**	***P*** [c]	**ES_w** [d]	**n**	**Mean (SD)**	**n**	**Mean (SD)**	***P*** [c]	**ES_w** [d]	**Mean (SD)**	***P*** [e]	**ES_b** [f]
EQ-5D VAS															
Baseline	314	85.8 (10.9)					326	86.3 (13.0)					-0.5 (12.0)	0.581	-0.04
Day 3	314	70.4 (16.9)	314	-15.4 (14.1)	0.000	-1.09	324	71.6 (16.9)	324	-14.7 (14.8)	0.000	-0.99	-0.7 (14.5)	0.544	-0.05
Week 2	266	81.6 (12.6)	265	-3.9 (11.0)	0.000	-0.35	258	83.0 (12.4)	258	-3.4 (11.1)	0.000	-0.31	-0.5 (11.0)	0.633	-0.04
Week 4	258	85.1 (11.3)	257	-0.4 (11.0)	0.526	-0.04	240	85.6 (11.6)	240	-0.6 (11.9)	0.433	-0.05	0.2 (11.4)	0.870	0.01
Utility Index															
Baseline	316	0.930 (0.116)					327	0.928 (0.104)					0.002 (0.110)	0.804	0.02
Day 3	316	0.822 (0.183)	316	-0.108 (0.159)	0.000	-0.68	327	0.817 (0.163)	327	-0.111 (0.152)	0.000	-0.73	0.002 (0.155)	0.859	0.01
Week 2	269	0.891 (0.142)	269	-0.040 (0.122)	0.000	-0.33	261	0.877 (0.150)	261	-0.052 (0.147)	0.000	-0.35	0.012 (0.135)	0.314	0.09
Week 4	260	0.904 (0.136)	260	-0.026 (0.127)	0.001	-0.21	246	0.896 (0.143)	246	-0.035 (0.132)	0.000	-0.26	0.008 (0.130)	0.476	0.06
Absenteeism															
Baseline	182	7.5 (20.1)					205	13.0 (27.3)					-5.5 (24.2)	0.027	-0.23
Week 1	196	51.2 (36.0)	178	43.1 (38.9)	0.000	1.11	224	62.7 (34.8)	200	49.7 (40.9)	0.000	1.22	-6.6 (39.9)	0.112	-0.16
Week 2	182	13.0 (24.8)	168	6.0 (30.3)	0.011	0.20	202	12.0 (25.2)	183	0.1 (36.6)	0.981	0.00	6.0 (33.7)	0.099	0.18

	**BNT162b2**						**Unvaccinated**						**Difference in Change from Baseline Between Cohorts**		
	**Score**		**Change from Baseline** [b]				**Score**		**Change from Baseline**						
Week 4	174	5.6 (15.0)	159	-1.8 (22.4)	0.304	-0.08	188	6.9 (16.3)	167	-5.2 (29.7)	0.025	-0.18	3.4 (26.4)	0.248	0.13
Presenteeism															
Baseline	179	11.7 (19.5)					197	16.0 (26.8)					-4.4 (23.6)	0.074	-0.18
Week 1	155	49.5 (29.2)	143	38.2 (28.7)	0.000	1.33	155	56.5 (30.9)	137	42.3 (33.4)	0.000	1.27	-4.1 (31.1)	0.273	-0.13
Week 2	174	19.8 (22.1)	159	7.3 (25.1)	0.000	0.29	196	21.6 (25.8)	172	7.3 (31.6)	0.003	0.23	0.0 (28.7)	0.993	0.00
Week 4	174	13.6 (21.0)	156	2.9 (25.2)	0.156	0.11	188	16.3 (22.0)	161	0.7 (28.9)	0.765	0.02	2.2 (27.2)	0.471	0.08
Productivity loss															
Baseline	179	16.1 (23.9)					197	20.4 (30.5)					-4.3 (27.6)	0.130	-0.16
Week 1	155	65.5 (27.9)	143	49.0 (32.9)	0.000	1.49	155	73.2 (27.5)	137	53.8 (35.2)	0.000	1.53	-4.8 (34.1)	0.239	-0.14
Week 2	174	25.5 (26.5)	159	8.6 (31.4)	0.001	0.27	196	26.5 (29.7)	172	7.4 (37.9)	0.012	0.19	1.2 (34.9)	0.750	0.04
Week 4	174	17.3 (24.7)	156	2.4 (29.8)	0.325	0.08	188	20.8 (26.2)	161	1.2 (35.2)	0.659	0.03	1.1 (32.6)	0.759	0.03
Activity impairment															
Baseline	284	14.2 (22.3)					281	18.5 (27.6)					-4.3 (25.1)	0.043	-0.17
Week 1	282	55.0 (29.2)	281	41.0 (33.7)	0.000	1.22	276	60.2 (29.3)	275	41.3 (35.1)	0.000	1.18	-0.3 (34.4)	0.905	-0.01
Week 2	269	22.8 (24.4)	268	8.1 (26.8)	0.000	0.30	261	23.9 (26.0)	260	6.7 (30.1)	0.000	0.22	1.4 (28.5)	0.581	0.05
Week 4	260	16.0 (22.0)	259	1.2 (26.0)	0.446	0.05	245	17.8 (23.0)	244	0.9 (28.7)	0.609	0.03	0.3 (27.4)	0.905	0.01
Hours missed															
Baseline	192	3.5 (10.2)					216	7.1 (16.0)					-3.6 (13.6)	0.008	-0.26

	BNT162b2						Unvaccinated						Difference in Change from Baseline Between Cohorts		
	Score		Change from Baseline [b]				Score		Change from Baseline						
Week 1	198	18.8 (14.7)	189	15.4 (16.4)	0.000	0.94	229	25.6 (17.5)	215	18.1 (18.9)	0.000	0.95	-2.7 (17.8)	0.131	-0.15
Week 2	189	5.2 (11.3)	182	1.6 (14.2)	0.140	0.11	208	5.2 (11.6)	196	-1.4 (18.7)	0.292	-0.08	3.0 (16.7)	0.085	0.18
Week 4	178	3.5 (11.6)	170	0.0 (12.4)	0.978	0.00	195	3.5 (9.8)	182	-2.9 (17.3)	0.023	-0.17	3.0 (15.1)	0.066	0.20
Hours worked															
Baseline	192	34.0 (16.2)					215	34.2 (17.1)					-0.2 (16.7)	0.883	-0.01
Week 1	198	19.3 (16.0)	189	-14.1 (17.6)	0.000	-0.80	228	15.3 (15.4)	213	-19.0 (21.3)	0.000	-0.89	4.9 (19.6)	0.013	0.25
Week 2	189	31.8 (15.4)	182	-2.1 (16.1)	0.078	-0.13	207	32.8 (14.6)	196	-1.8 (19.1)	0.177	-0.10	-0.3 (17.7)	0.882	-0.02
Week 4	178	34.8 (13.7)	170	1.0 (17.2)	0.459	0.06	195	35.1 (14.0)	182	0.3 (18.0)	0.795	0.02	0.6 (17.6)	0.736	0.04
PROMIS Fatigue [g]															
Baseline	283	44.0 (8.9)					281	45.4 (10.0)					-1.5 (9.4)	0.065	-0.16
Week 1	282	59.6 (9.7)	280	15.7 (11.0)	0.000	1.43	277	60.8 (9.9)	276	15.3 (12.0)	0.000	1.28	0.3 (11.5)	0.727	0.03
Week 2	269	51.0 (10.0)	267	6.8 (10.2)	0.000	0.66	261	51.3 (10.2)	260	6.0 (12.0)	0.000	0.51	0.7 (11.1)	0.442	0.07
Week 4	260	46.9 (10.2)	258	2.8 (9.2)	0.000	0.30	246	48.3 (10.1)	245	3.1 (11.2)	0.000	0.28	-0.3 (10.2)	0.728	-0.03

[a] Score ranges: EQ-5D-5L VAS 0 to 100, EQ-5D-5L UI (the United States weights) -0.573 to 1; WPAI-GH (absenteeism, presenteeism, work productivity loss, and activity impairment) 0 to 100 percent. [b] Baseline refers to pre-COVID-19 symptom onset. [c] P value of t-test comparing mean score changes from baseline and 0 within BNT162b2 or Unvaccinated cohorts. [d] ESw refers to the standardized effect size for score changes from baseline within BNT162b2 or Unvaccinated cohorts. [e] P value of t-test comparing mean score changes from baseline between BNT162b2 and Unvaccinated cohorts. [f] ESb refers to the standardized effect size for score changes from baseline between BNT162b2 and Unvaccinated cohorts. [g] Higher values indicate more fatigue.

Table S5 Least-Square Mean Estimates of EQ-5D-5L, PROMIS Fatigue and WPAI-GH Scores and Their Changes from Pre-COVID-19 Baseline by Assessment Time and Vaccination Status

	**BNT162b2 Cohort**				**Unvaccinated Cohort**				**Between Cohort Difference**		
	**Score**	**Change from Baseline**			**Score**	**Change from Baseline**					
**Variable**	**LSE (95% CI)**	**LSE (95% CI)**	***P*** [b]	**ES_w** [c]	**LSE (95% CI)**	**LSE (95% CI)**	***P*** [b]	**ES_w** [c]	**LSE (95% CI)**	***P*** [d]	**ES_b** [e]
EQ VAS											
Day 3	69.7 (66.6, 72.8)	-16.3 (-19.4, 013.1)	0.000	-1.15	70.5 (67.3, 73.7)	-15.4 (-18.6, -12.2)	0.000	-1.04	-0.8 (-3.1, 1.4)	0.470	-0.06
Week 2	80.9 (78.0, 83.9)	-5.0 (-8.0, -2.1)	0.001	-0.46	82.0 (78.9, 85.1)	-3.9 (-7.0, -0.9)	0.012	-0.36	-1.1 (-2.9, 0.7)	0.242	-0.10
Week 4	84.5 (81.5, 87.4)	-1.5 (-4.4, 1.5)	0.332	-0.13	84.7 (81.6, 87.8)	-1.2 (-4.3, 1.8)	0.427	-0.10	-0.2 (-2.0, 1.6)	0.815	-0.02
Utility Index (US weight)											
Day 3	0.744 (0.708, 0.781)	-0.185 (-0.221, -0.149)	0.000	-1.17	0.746 (0.709, 0.783)	-0.183 (-0.220, -0.146)	0.000	-1.21	-0.002 (-0.026, 0.022)	0.861	-0.01
Week 2	0.812 (0.776, 0.847)	-0.118 (-0.153, -0.082)	0.000	-0.96	0.806 (0.769, 0.843)	-0.124 (-0.160, -0.087)	0.000	-0.84	0.006 (-0.016, 0.028)	0.593	0.04
Week 4	0.825 (0.790, 0.861)	-0.104 (-0.140, -0.069)	0.000	-0.82	0.824 (0.787, 0.860)	-0.106 (-0.143, -0.069)	0.000	-0.80	0.002 (-0.020, 0.023)	0.885	0.01
Absenteeism											
Week 1	52.0 (43.6, 60.5)	42.6 (34.1, 51.1)	0.000	1.10	64.2 (55.6, 72.9)	54.8 (46.1, 63.4)	0.000	1.34	-12.2 (-19.4, -5.0)	0.001	-0.31
Week 2	14.4 (6.6, 22.1)	4.9 (-2.8, 12.7)	0.212	0.16	13.3 (5.3, 21.4)	3.9 (-4.1, 11.9)	0.343	0.11	1.0 (-4.3, 6.4)	0.698	0.03
Week 4	6.9 (-0.4, 14.1)	-2.6 (-9.8, 4.6)	0.479	-0.12	7.7 (0.2, 15.3)	-1.7 (-9.3, 5.8)	0.655	-0.06	-0.9 (-4.3, 2.6)	0.616	-0.03
Presenteeism											

	BNT162b2 Cohort				Unvaccinated Cohort				Between Cohort Difference		
	Score	Change from Baseline			Score	Change from Baseline					
Week 1	51.1 (40.6, 61.6)	37.7 (27.2, 48.3)	0.000	1.31	56.8 (45.9, 67.6)	43.4 (32.5, 54.2)	0.000	1.30	-5.6 (-12.3, 1.0)	0.095	-0.18
Week 2	19.4 (9.3, 29.6)	6.1 (-4.1, 16.2)	0.240	0.24	21.2 (10.7, 31.6)	7.8 (-2.7, 18.2)	0.146	0.25	-1.7 (-6.9, 3.4)	0.514	-0.06
Week 4	14.6 (4.6, 24.6)	1.2 (-8.8, 11.2)	0.808	0.05	14.4 (4.0, 24.8)	1.0 (-9.4, 11.4)	0.852	0.03	0.3 (-4.5, 5.0)	0.917	0.01
Work productivity loss											
Week 1	62.9 (51.4, 74.4)	45.3 (33.8, 56.8)	0.000	1.38	68.8 (57.0, 80.7)	51.2 (39.3, 63.1)	0.000	1.45	-5.9 (-12.3, 0.5)	0.069	-0.17
Week 2	21.4 (10.0, 32.8)	3.8 (-7.7, 15.2)	0.518	0.12	22.1 (10.3, 33.9)	4.5 (-7.3, 16.3)	0.456	0.12	-0.7 (-6.8, 5.4)	0.816	-0.02
Week 4	14.5 (3.2, 25.8)	-3.1 (-14.4, 8.2)	0.587	-0.10	15.4 (3.7, 27.1)	-2.3 (-14.0, 9.5)	0.706	-0.06	-0.9 (-6.5, 4.8)	0.762	-0.03
Activity impairment											
Week 1	58.9 (52.3, 65.4)	42.8 (36.2, 49.3)	0.000	1.27	61.5 (54.8, 68.2)	45.4 (38.7, 52.1)	0.000	1.29	-2.6 (-7.4, 2.2)	0.285	-0.08
Week 2	26.2 (19.9, 32.5)	10.1 (3.8, 16.4)	0.002	0.38	25.6 (19.0, 32.1)	9.5 (2.9, 16.0)	0.005	0.31	0.6 (-3.5, 4.8)	0.760	0.02
Week 4	19.7 (13.5, 25.9)	3.6 (-2.6, 9.8)	0.258	0.14	19.8 (13.3, 26.3)	3.7 (-2.8, 10.2)	0.264	0.13	-0.1 (-4.0, 3.8)	0.959	0.00
Hours missed											
Week 1	18.0 (13.9, 22.2)	13.1 (8.9, 17.3)	0.000	0.80	24.2 (19.9, 28.4)	19.2 (15.0, 23.5)	0.000	1.02	-6.1 (-9.3, -3.0)	0.000	-0.34
Week 2	4.4 (0.5, 8.2)	-0.6 (-4.4, 3.3)	0.776	-0.04	4.0 (0.0, 8.0)	-0.9 (-5.0, 3.1)	0.644	-0.05	0.4 (-2.0, 2.7)	0.749	0.02
Week 4	2.6 (-1.3, 6.5)	-2.3 (-6.2, 1.5)	0.238	-0.19	2.3 (-1.7, 6.3)	-2.6 (-6.6, 1.4)	0.200	-0.15	0.3 (-2.0, 2.6)	0.807	0.02
Hours worked											
Week 1	16.2 (11.4, 21.1)	-18.3 (-23.1, -13.4)	0.000	-1.04	11.7 (6.7, 16.6)	-22.8 (-27.8, -17.9)	0.000	-1.07	4.6 (1.5, 7.6)	0.003	0.23
Week 2	28.7 (23.9, 33.5)	-5.8 (-10.6, -1.0)	0.019	-0.36	29.7 (24.7, 34.6)	-4.8 (-9.8, 0.1)	0.056	-0.25	-0.9 (-3.8, 1.9)	0.512	-0.05
Week 4	31.6 (26.8, 36.4)	-2.9 (-7.7, 1.9)	0.239	-0.17	31.8 (26.9, 36.8)	-2.7 (-7.6, 2.3)	0.289	-0.15	-0.2 (-3.0, 2.6)	0.878	-0.01

	BNT162b2 Cohort				Unvaccinated Cohort				Between Cohort Difference		
	Score	Change from Baseline			Score	Change from Baseline					
PROMIS Fatigue [g]											
Week 1	61.7 (59.2, 64.3)	17.1 (14.5, 19.6)	0.000	1.56	62.0 (59.4, 64.6)	17.3 (14.7, 20.0)	0.000	1.44	-0.3 (-1.8, 1.3)	0.750	-0.02
Week 2	53.0 (50.4, 55.6)	8.3 (5.8, 10.9)	0.000	0.81	52.2 (49.6, 54.9)	7.6 (4.9, 10.2)	0.000	0.63	0.8 (-0.8, 2.4)	0.347	0.07
Week 4	49.0 (46.4, 51.6)	4.3 (1.7, 6.9)	0.001	0.47	49.2 (46.5, 51.9)	4.5 (1.8, 7.2)	0.001	0.40	-0.2 (-1.9, 1.4)	0.785	-0.02

VAS=Visual Analogue Scale; LSE = least-square estimate; CI = confidence interval. [a] Multivariate models include variables for time, vaccination status and interaction of time by vaccination status, as well as covariates of participant pre-COVID-19 symptom onset score, sociodemographic characteristics (age, sex, regions, social vulnerability, race/ethnicity), variable for at least 1 comorbidity, previously tested positive for COVID-19, severity of acute illness (number of symptoms reported on index date), and prescription of Paxlovid. [b] Pvalue refers to the comparison of lease-square mean estimates score changes from baseline and 0 within BNT162b2 or Unvaccinated cohorts. [c] ESw, within-cohort effect size, was calculated as the least square estimate of mean change from divided by the observed standard deviation of change scores from baseline to follow-up. [d] Pvalue refers to the difference in lease-square mean estimates between BNT162b2 and Unvaccinated cohorts. [e] ESb, between-cohort effect size, was calculated as the difference in least square estimates of mean changes from baseline between cohorts, divided by the observed pooled standard deviation of change scores. [g] Higher values indicate more fatigue.

Table S6 Mixed Models for Repeated Measurements EQ-5D-5L and WPAI-GH Scores: Estimate (Standard Error)

	EQ VAS	EQ-5D-5L UI	Absenteeism	Presenteeism	Work Productivity Loss	Activity Impairment	Hours Missed due to health	Actual hours worked	PROMIS Fatigue 8(a)
Intercept	25.0 (3.4)	0.310 (0.043)	3.6 (3.7)	0.4 (4.8)	6.3 (5.3)	1.9 (3.8)	-0.2 (2.1)	24.3 (2.8)	27.1 (2.1)
Time									
Day 3 / Week 1	Ref	Ref	Ref	Ref	Ref	Ref	Ref	Ref	Ref
Week 2	11.5 (0.8)	0.059 (0.009)	56.5 (2.7)	42.4 (2.7)	53.5 (2.8)	41.7 (2.1)	21.9 (1.3)	-20.2 (1.3)	12.8 (0.6)
Week 4	14.2 (0.9)	0.077 (0.009)	5.6 (2.0)	6.8 (1.6)	6.7 (2.0)	5.8 (1.4)	1.7 (1.1)	-2.2 (1.1)	3.0 (0.5)
BNT162b2									
No	Ref	Ref	Ref	Ref	Ref	Ref	Ref	Ref	Ref
Yes	-0.8 (1.2)	-0.002 (0.012)	-0.9 (1.8)	0.3 (2.4)	-0.9 (2.9)	-0.1 (2.0)	0.3 (1.2)	-0.2 (1.4)	-0.2 (0.8)
BNT162b2by Time									
Yes * Day 3/Week 1	Ref	Ref	Ref	Ref	Ref	Ref	Ref	Ref	Ref
Yes * Week 2	-0.2 (1.1)	0.008 (0.013)	-11.3 (3.8)	-5.9 (3.8)	-5.1 (4.0)	-2.5 (2.9)	-6.4 (1.9)	4.8 (1.9)	0.0 (0.9)
Yes * Week 4	0.6 (1.3)	0.004 (0.013)	1.9 (2.9)	-2.0 (2.3)	0.1 (2.9)	0.7 (2.0)	0.1 (1.5)	-0.7 (1.6)	1.0 (0.7)
Baseline score	0.6 (0.0)	0.624 (0.039)	0.0 (0.0)	0.3 (0.0)	0.2 (0.0)	0.3 (0.0)	0.1 (0.0)	0.3 (0.0)	0.3 (0.0)
Age group									
18-29	Ref	Ref	Ref	Ref	Ref	Ref	Ref	Ref	Ref
30-49	-0.7 (1.1)	-0.010 (0.013)	-0.1 (2.1)	6.0 (2.7)	5.3 (3.0)	4.7 (2.2)	0.8 (1.1)	2.6 (1.5)	2.3 (0.9)
50-64	-1.6 (1.2)	-0.016 (0.015)	3.3 (2.4)	4.4 (3.2)	5.0 (3.5)	3.5 (2.5)	1.7 (1.3)	1.9 (1.7)	2.0 (1.1)
65-74	0.1 (1.4)	-0.005 (0.017)	1.0 (3.8)	-2.9 (5.0)	-5.6 (5.5)	-1.4 (2.9)	-0.6 (2.1)	-3.3 (2.7)	0.9 (1.2)
75+	-0.1 (2.3)	0.007 (0.028)	-1.5 (9.5)	9.2 (11.6)	4.9 (12.5)	0.2 (4.8)	-3.5 (4.5)	-0.6 (5.7)	0.3 (2.0)
Gender									
Female	Ref	Ref	Ref	Ref	Ref	Ref	Ref	Ref	Ref
Male	1.6 (0.8)	0.016 (0.010)	-0.2 (1.7)	-1.8 (2.3)	-0.9 (2.5)	-4.3 (1.7)	1.3 (1.0)	3.1 (1.2)	-3.1 (0.7)
Unknown	-5.2 (4.2)	-0.224 (0.050)	3.6 (9.1)	6.9 (13.2)	-1.6 (15.0)	11.7 (8.7)	-0.1 (4.8)	-10.0 (6.0)	7.5 (3.6)
Race/Ethnicity									

	EQ VAS	EQ-5D-5L UI	Absenteeism	Presenteeism	Work Productivity Loss	Activity Impairment	Hours Missed due to health	Actual hours worked	PROMIS Fatigue 8(a)
White or Caucasian	Ref	Ref	Ref	Ref	Ref	Ref	Ref	Ref	Ref
Black or African American	3.7 (1.3)	0.028 (0.016)	1.3 (2.9)	-2.5 (3.7)	-0.6 (4.0)	-2.1 (2.8)	1.4 (1.5)	0.9 (2.0)	-1.8 (1.2)
Hispanic	1.1 (1.1)	-0.023 (0.014)	3.5 (2.3)	0.3 (3.0)	2.3 (3.4)	2.6 (2.4)	1.4 (1.2)	-2.8 (1.6)	0.6 (1.0)
Asian	1.5 (1.3)	0.022 (0.016)	-1.3 (2.6)	-5.9 (3.4)	-6.8 (3.8)	-4.0 (2.7)	0.1 (1.5)	0.1 (1.9)	-1.4 (1.1)
Patient Refused	0.2 (2.1)	-0.030 (0.025)	7.7 (4.3)	0.5 (5.8)	3.0 (6.6)	8.3 (4.3)	3.2 (2.3)	2.4 (3.0)	0.3 (1.8)
Other	1.7 (1.8)	0.000 (0.022)	-2.4 (3.8)	-9.4 (5.2)	-11.3 (5.6)	-1.3 (3.7)	-1.9 (2.1)	3.5 (2.7)	-0.6 (1.6)
US Region									
Northeast	Ref	Ref	Ref	Ref	Ref	Ref	Ref	Ref	Ref
South	0.8 (1.2)	0.026 (0.014)	-0.1 (2.4)	-0.7 (3.1)	-1.1 (3.4)	-1.7 (2.4)	-0.5 (1.3)	-0.1 (1.7)	-1.1 (1.0)
Midwest	-1.2 (1.2)	-0.002 (0.015)	-0.7 (2.5)	-1.1 (3.2)	-0.5 (3.5)	-0.7 (2.6)	-1.1 (1.3)	0.9 (1.7)	-1.2 (1.1)
West	0.9 (1.3)	0.019 (0.015)	0.5 (2.6)	-0.4 (3.3)	-0.1 (3.6)	-2.8 (2.6)	0.5 (1.4)	1.3 (1.8)	-1.1 (1.1)
Social Vulnerability Index									
<0.25	Ref	Ref	Ref	Ref	Ref	Ref	Ref	Ref	Ref
≥0.25 and <0.5	-1.0 (0.9)	-0.003 (0.012)	1.3 (2.0)	-1.1 (2.6)	-0.6 (2.8)	0.7 (1.9)	1.3 (1.1)	-1.9 (1.4)	0.0 (0.8)
≥0.5 and <0.75	-0.3 (1.1)	-0.008 (0.013)	0.0 (2.2)	-1.3 (2.9)	-2.0 (3.2)	-0.2 (2.2)	0.0 (1.2)	-1.9 (1.6)	-0.3 (0.9)
≥0.75	-1.0 (1.3)	-0.018 (0.016)	0.9 (2.8)	-3.6 (3.6)	-3.1 (4.0)	-1.3 (2.7)	0.5 (1.5)	-0.9 (2.0)	0.6 (1.1)
≥1 Comorbidity	-0.9 (0.9)	-0.013 (0.010)	-0.4 (1.8)	3.2 (2.3)	2.9 (2.5)	2.2 (1.8)	0.9 (1.0)	-1.5 (1.2)	0.2 (0.7)
Previously tested positive	1.2 (0.7)	0.006 (0.009)	-0.7 (1.5)	-3.0 (1.9)	-4.7 (2.1)	-3.6 (1.5)	-0.7 (0.8)	-0.8 (1.0)	-1.2 (0.6)
Number of Acute COVID Symptoms on index day	-0.7 (0.2)	-0.013 (0.002)	0.1 (0.3)	1.9 (0.4)	1.8 (0.5)	1.9 (0.3)	0.1 (0.2)	0.2 (0.2)	1.1 (0.1)
Paxlovid prescription	-1.1 (0.9)	-0.016 (0.011)	0.1 (1.9)	0.5 (2.4)	0.8 (2.7)	4.2 (1.8)	1.1 (1.0)	1.0 (1.3)	2.0 (0.8)

Table S7. EQ-5D-5L and WPAI-GH Scores post-matching

	BNT162b2						Unvaccinated						Difference in Change from Baseline Between Cohorts		
	Score		Change from Baseline [b]				Score		Change from Baseline						
	n	Mean (SD)	n	Mean (SD)	*P* [c]	ES_w [d]	n	Mean (SD)	n	Mean (SD)	*P* [c]	ES_w [d]	Mean (SD)	*P* [e]	ES_b [f]
EQ VAS															
Baseline	314	85.4 (10.8)					326	86.4 (12.5)					-0.9 (11.7)	0.307	-0.08
Day 3	314	70.1 (16.4)	314	-15.3 (13.7)	0.000	-1.12	324	71.5 (17.0)	324	-14.9 (15.1)	0.000	-0.99	-0.4 (14.4)	0.731	-0.03
Week 2	266	81.7 (12.2)	265	-3.6 (10.8)	0.000	-0.33	258	83.1 (11.7)	258	-3.7 (10.5)	0.000	-0.35	0.1 (10.7)	0.921	0.01
Week 4	258	85.2 (11.0)	257	-0.2 (10.3)	0.777	-0.02	240	85.6 (11.0)	240	-1.2 (11.5)	0.122	-0.10	1.0 (10.9)	0.322	0.09
Utility Index															
Baseline[e]	316	0.926 (0.116)					327	0.928 (0.104)					-0.002 (0.110)	0.840	-0.02
Day 3	316	0.816 (0.188)	316	-0.110 (0.155)	0.000	-0.71	327	0.820 (0.162)	327	-0.108 (0.152)	0.000	-0.71	-0.002 (0.154)	0.879	-0.01
Week 2	269	0.891 (0.140)	269	-0.036 (0.119)	0.000	-0.31	261	0.883 (0.140)	261	-0.050 (0.138)	0.000	-0.37	0.014 (0.129)	0.212	0.11
Week 4	260	0.904 (0.137)	260	-0.022 (0.125)	0.004	-0.18	246	0.898 (0.133)	246	-0.038 (0.126)	0.000	-0.30	0.016 (0.125)	0.161	0.12
Absenteeism															
Baseline[e]	182	7.3 (20.7)					205	11.4 (25.1)					-4.1 (23.1)	0.084	-0.18
Week 1	196	51.0 (37.3)	178	42.8 (39.6)	0.000	1.08	224	62.8 (33.8)	200	51.3 (40.1)	0.000	1.28	-8.5 (39.9)	0.039	-0.21
Week 2	182	11.6 (23.8)	168	4.8 (29.3)	0.036	0.16	202	12.5 (24.0)	183	1.7 (33.5)	0.488	0.05	3.1 (31.6)	0.367	0.10
Week 4	174	5.3 (15.2)	159	-2.5 (24.6)	0.201	-0.10	188	6.4 (15.1)	167	-4.8 (27.0)	0.022	-0.18	2.3 (25.9)	0.420	0.09
Presenteeism															
Baseline[e]	179	12.0 (20.5)					197	15.4 (25.2)					-3.3 (23.1)	0.163	-0.14
Week 1	155	49.1 (30.2)	143	37.7 (29.3)	0.000	1.29	155	56.5 (30.2)	137	42.9 (30.9)	0.000	1.39	-5.2 (30.1)	0.150	-0.17
Week 2	174	20.0 (23.3)	159	7.6 (26.0)	0.000	0.29	196	21.5 (23.9)	172	8.2 (29.1)	0.000	0.28	-0.5 (27.7)	0.865	-0.02
Week 4	174	13.3 (21.7)	156	2.5 (26.7)	0.236	0.10	188	16.1 (20.5)	161	1.6 (27.0)	0.447	0.06	0.9 (26.8)	0.761	0.03
Work productivity loss															
Baseline[e]	179	16.0 (24.4)					197	19.3 (28.7)					-3.3 (26.7)	0.231	-0.12
Week 1	155	65.0 (28.5)	143	48.9 (32.9)	0.000	1.49	155	73.7 (26.5)	137	54.6 (33.0)	0.000	1.65	-5.7 (33.0)	0.151	-0.17
Week 2	174	25.2 (27.6)	159	8.6 (31.8)	0.001	0.27	196	27.2 (27.9)	172	9.8 (35.7)	0.000	0.27	-1.2 (33.9)	0.753	-0.03

	BNT162b2						Unvaccinated						Difference in Change from Baseline Between Cohorts		
	Score		Change from Baseline [b]				Score		Change from Baseline						
	n	Mean (SD)	n	Mean (SD)	*P* [c]	ES_w [d]	n	Mean (SD)	n	Mean (SD)	*P* [c]	ES_w [d]	Mean (SD)	*P* [e]	ES_b [f]
Week 4	174	16.9 (25.5)	156	2.0 (31.1)	0.411	0.07	188	20.1 (24.4)	161	2.0 (32.4)	0.439	0.06	0.1 (31.7)	0.985	0.00
Activity impairment															
Baseline[e]	284	14.0 (22.0)					281	18.5 (27.8)					-4.5 (25.0)	0.033	-0.18
Week 1	282	55.5 (29.2)	281	41.7 (33.3)	0.000	1.25	276	59.6 (29.0)	275	40.6 (35.5)	0.000	1.15	1.0 (34.4)	0.723	0.03
Week 2	269	22.8 (24.6)	268	8.4 (26.9)	0.000	0.31	261	24.4 (25.8)	260	6.6 (29.0)	0.000	0.23	1.8 (27.9)	0.451	0.07
Week 4	260	15.3 (21.2)	259	0.8 (26.2)	0.623	0.03	245	18.7 (23.9)	244	1.0 (27.6)	0.580	0.04	-0.2 (26.9)	0.941	-0.01
Hours missed due to health															
Baseline[e]	192	3.4 (10.2)					216	6.1 (14.4)					-2.8 (12.6)	0.027	-0.22
Week 1	198	18.7 (15.1)	189	15.3 (17.0)	0.000	0.90	229	25.4 (16.7)	215	18.9 (18.4)	0.000	1.03	-3.6 (17.7)	0.043	-0.20
Week 2	189	4.6 (11.0)	182	1.0 (14.2)	0.342	0.07	208	5.4 (11.2)	196	-0.5 (17.2)	0.706	-0.03	1.5 (15.8)	0.368	0.09
Week 4	178	3.3 (11.2)	170	-0.4 (12.8)	0.706	-0.03	195	3.1 (8.6)	182	-2.8 (15.6)	0.015	-0.18	2.5 (14.3)	0.108	0.17
Hours worked															
Baseline[e]	192	34.4 (16.8)					215	33.8 (16.0)					0.6 (16.4)	0.721	0.04
Week 1	198	19.2 (16.3)	189	-14.4 (18.1)	0.000	-0.79	228	15.2 (14.9)	213	-18.9 (20.6)	0.000	-0.92	4.5 (19.5)	0.020	0.23
Week 2	189	31.6 (16.0)	182	-2.4 (16.4)	0.049	-0.15	207	33.3 (13.6)	196	-1.4 (17.4)	0.245	-0.08	-1.0 (16.9)	0.584	-0.06
Week 4	178	34.8 (13.6)	170	0.9 (16.7)	0.475	0.05	195	35.7 (13.3)	182	0.8 (16.8)	0.540	0.05	0.2 (16.7)	0.933	0.01
PROMIS Fatigue 8(a)															
Baseline[e]	283	44.6 (8.9)					281	45.1 (9.9)					-0.5 (9.4)	0.560	-0.05
Week 1	282	59.9 (9.6)	280	15.5 (11.1)	0.000	1.40	277	60.5 (10.0)	276	15.3 (11.6)	0.000	1.31	0.2 (11.4)	0.849	0.02
Week 2	269	50.9 (10.0)	267	6.3 (10.3)	0.000	0.61	261	51.1 (10.0)	260	6.2 (11.5)	0.000	0.53	0.1 (10.9)	0.899	0.01
Week 4	260	46.9 (10.1)	258	2.3 (9.4)	0.000	0.24	246	48.1 (10.1)	245	3.1 (10.9)	0.000	0.28	-0.8 (10.2)	0.386	-0.08

[a] Score ranges: EQ-5D-5L VAS 0 to 100, EQ-5D-5L UI (the United States weights) -0.573 to 1; WPAI-GH (absenteeism, presenteeism, work productivity loss, and activity impairment) 0 to 100 percent.

[b] Baseline refers to pre-COVID-19 symptom onset.

[c] P-value of t-test comparing mean score changes from baseline and 0 within BNT162b2 or Unvaccinated cohorts.

[d] ES_w refers to the standardized effect size for score changes from baseline within BNT162b2 or Unvaccinated cohorts.

[e] P-value of t-test comparing mean score changes from baseline between BNT162b2 and Unvaccinated cohorts.

[f] ES_b refers to the standardized effect size for score changes from baseline between BNT162b2 and Unvaccinated cohorts.

Figure S1 SARS-CoV-2 Variants proportions in the US

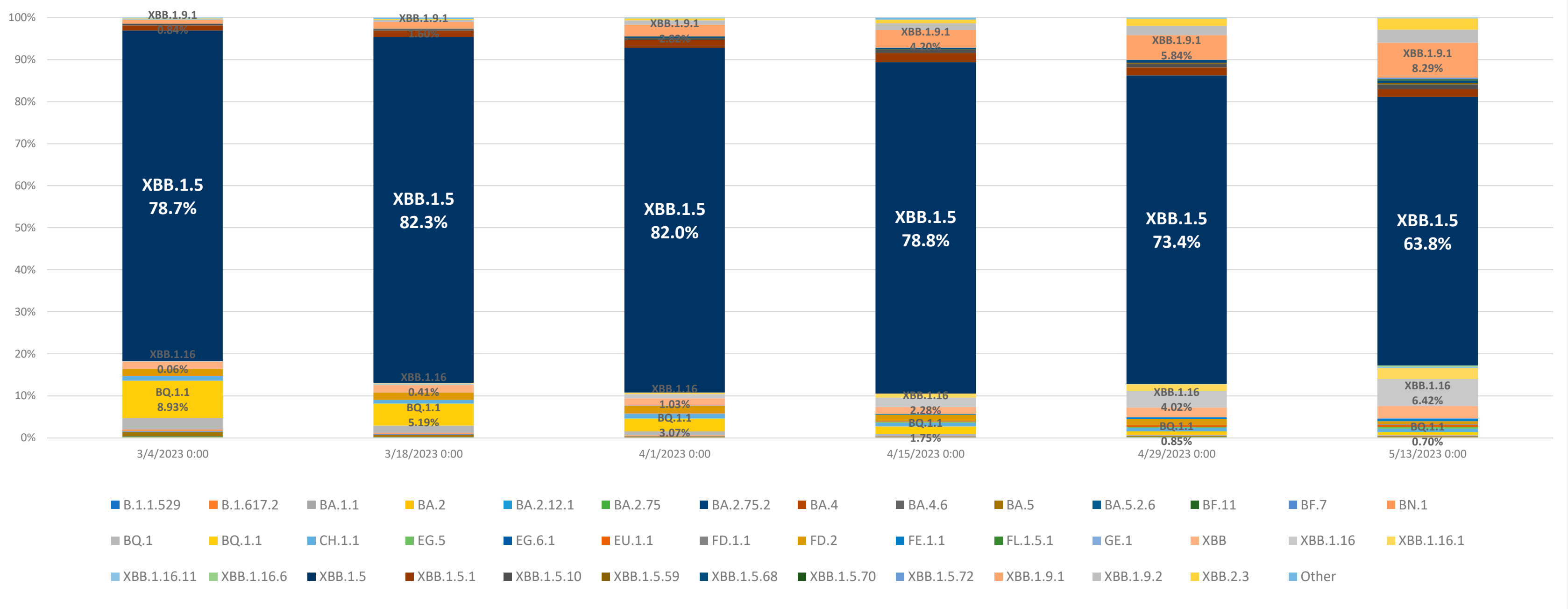

Source: reproduced and adapted from SARS-CoV-2 Variant Proportions | Data | Centers for Disease Control and Prevention (cdc.gov).

An analysis of CDC variants data revealed that XBB.1.5 was predominant throughout the study period, ranging from 63.8% - 82.3%. BQ.1.1 was up to 8.9% in early March, and fell throughout April. XBB.1.16 and XBB.1.9.1 increased throughout April/May to 6-10% of cases.

Figure S2 Prevalence of acute COVID-19 symptoms by vaccination status

(a) Time of testing

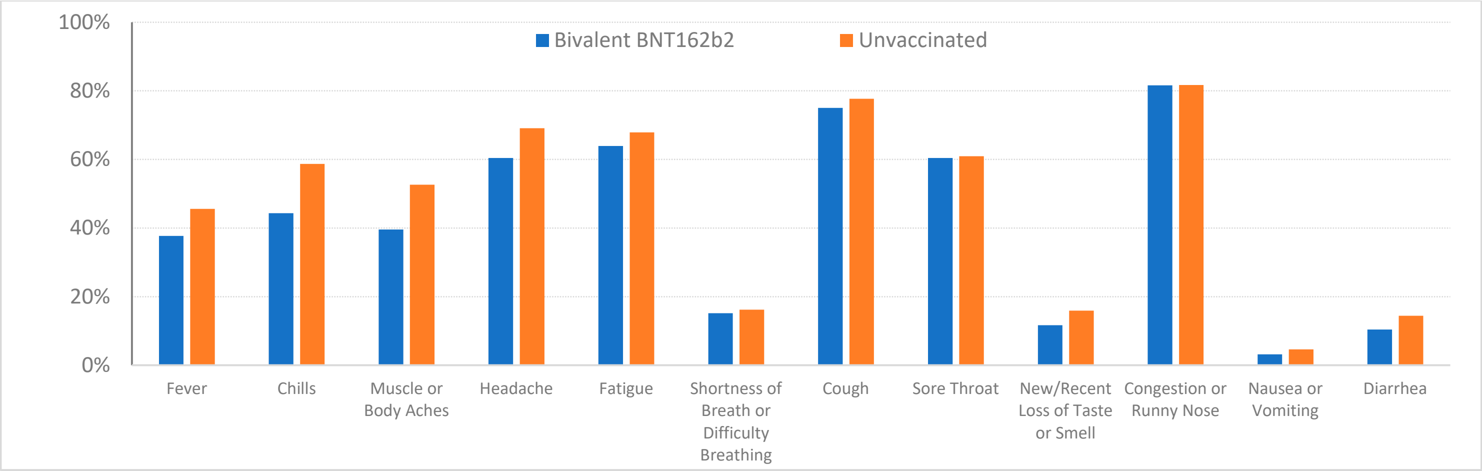

(b) Week 1

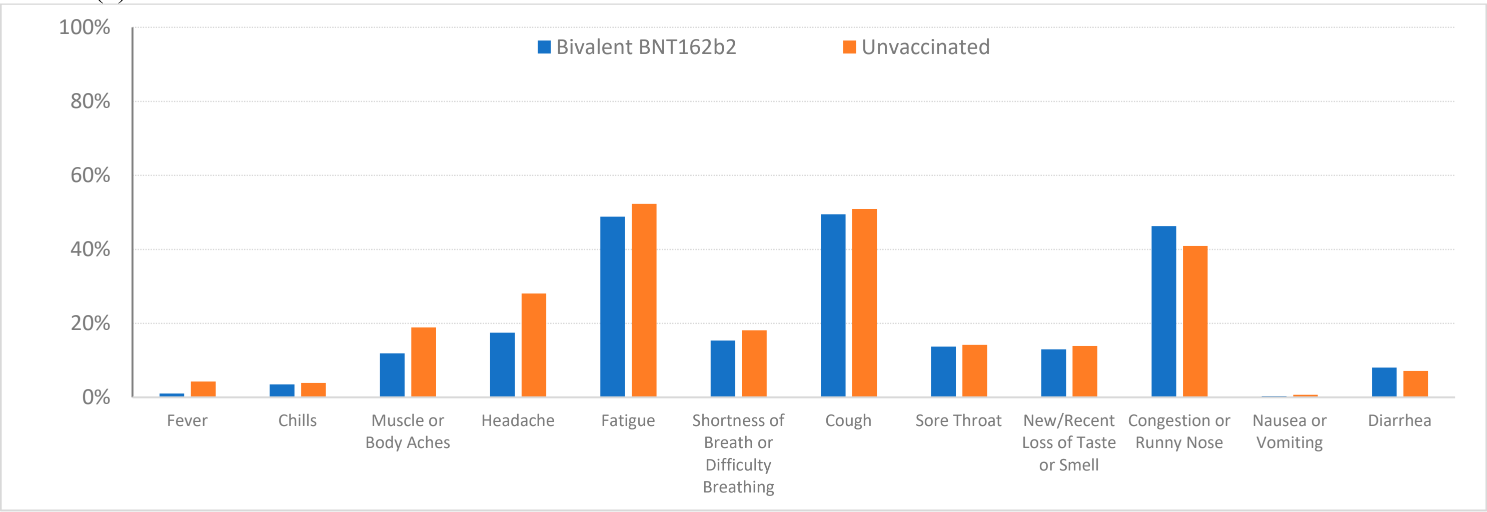

(c) Week 2

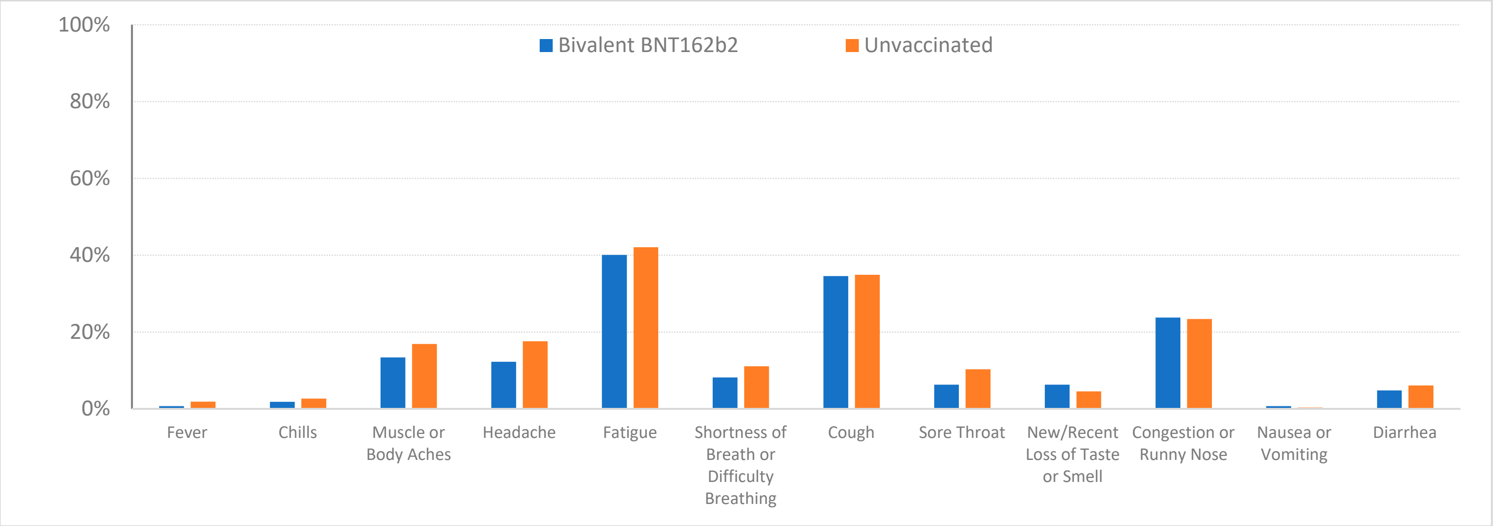

100%
80%
60%
40%
20%
0%
Bivalent BNT162b2
Unvaccinated
Fever
Chills
Muscle or Body Aches
Headache
Fatigue
Shortness of Breath or Difficulty Breathing
Cough
Sore Throat
New/Recent Loss of Taste or Smell
Congestion or Runny Nose
Nausea or Vomiting
Diarrhea

(d) Week 4

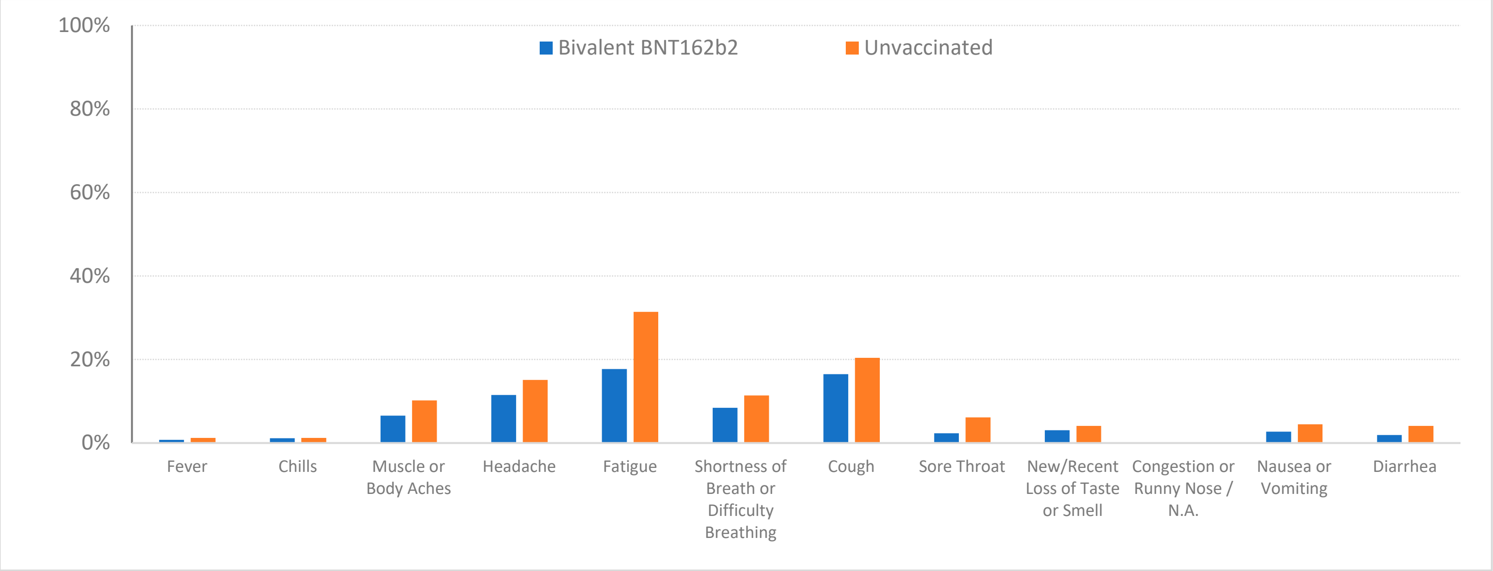

100%
80%
60%
40%
20%
0%
Bivalent BNT162b2
Unvaccinated
Fever
Chills
Muscle or Body Aches
Headache
Fatigue
Shortness of Breath or Difficulty Breathing
Cough
Sore Throat
New/Recent Loss of Taste or Smell
Congestion or Runny Nose / N.A.
Nausea or Vomiting
Diarrhea

Figure S3 Prevalence of acute COVID-19 symptoms over time by category

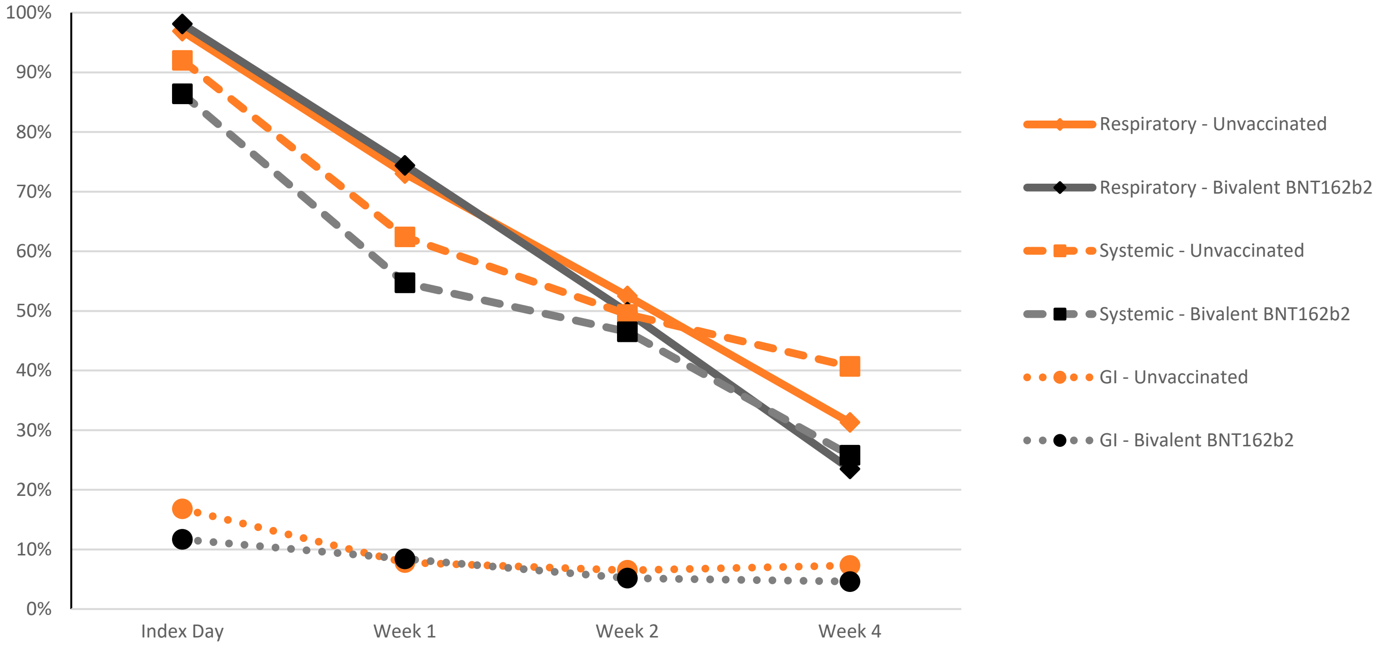

Supplement: Supplementary file 1 [file vaccines-11-01669-s001.zip › vaccines-2649612-supplementary.pdf]
